# Supplementary material for: ‘I actually felt like I was a researcher myself.’ On involving children in the analysis of qualitative paediatric research in the Netherlands
Source: BMJ Open. 2020 Aug 30;10(8):e034433. doi: 10.1136/bmjopen-2019-034433 (PMC7462149; doi:10.1136/bmjopen-2019-034433)
Supplement: Author's manuscript [file bmjopen-2019-034433.draft_revisions.pdf]

BMJ Open

BMJ Open is committed to open peer review. As part of this commitment we make the peer review history of every article we publish publicly available.

When an article is published we post the peer reviewers' comments and the authors' responses online. We also post the versions of the paper that were used during peer review. These are the versions that the peer review comments apply to.

The versions of the paper that follow are the versions that were submitted during the peer review process. They are not the versions of record or the final published versions. They should not be cited or distributed as the published version of this manuscript.

BMJ Open is an open access journal and the full, final, typeset and author-corrected version of record of the manuscript is available on our site with no access controls, subscription charges or pay-per-view fees (http://bmjopen.bmj.com).

If you have any questions on BMJ Open's open peer review process please email info.bmjopen@bmj.com

BMJ Open

"I actually felt like I was a researcher myself": Involving Children as Co-Researchers in Analysis of Qualitative Paediatric Research

Journal:	*BMJ Open*
Manuscript ID	bmjopen-2019-034433
Article Type:	Original research
Date Submitted by the Author:	20-Sep-2019
Complete List of Authors:	Luchtenberg, Malou; University of Groningen; University Medical Center Groningen, Department of Pediatrics<br>Maeckelberghe, Els; University of Groningen, Institute for Medical Education; University Medical Center Groningen<br>Verhagen, Eduard; University of Groningen; University Medical Center Groningen , Department of Pediatrics
Keywords:	Involvement, Children, PAEDIATRICS, Patient Participation, Biomedical Research, QUALITATIVE RESEARCH

SCHOLARONE™
Manuscripts

I, the Submitting Author has the right to grant and does grant on behalf of all authors of the Work (as defined in the below author licence), an exclusive licence and/or a non-exclusive licence for contributions from authors who are: i) UK Crown employees; ii) where BMJ has agreed a CC-BY licence shall apply, and/or iii) in accordance with the terms applicable for US Federal Government officers or employees acting as part of their official duties; on a worldwide, perpetual, irrevocable, royalty-free basis to BMJ Publishing Group Ltd ("BMJ") its licensees and where the relevant Journal is co-owned by BMJ to the co-owners of the Journal, to publish the Work in this journal and any other BMJ products and to exploit all rights, as set out in our licence.

The Submitting Author accepts and understands that any supply made under these terms is made by BMJ to the Submitting Author unless you are acting as an employee on behalf of your employer or a postgraduate student of an affiliated institution which is paying any applicable article publishing charge ("APC") for Open Access articles. Where the Submitting Author wishes to make the Work available on an Open Access basis (and intends to pay the relevant APC), the terms of reuse of such Open Access shall be governed by a Creative Commons licence – details of these licences and which Creative Commons licence will apply to this Work are set out in our licence referred to above.

Other than as permitted in any relevant BMJ Author's Self Archiving Policies, I confirm this Work has not been accepted for publication elsewhere, is not being considered for publication elsewhere and does not duplicate material already published. I confirm all authors consent to publication of this Work and authorise the granting of this licence.

For peer review only

“I actually felt like I was a researcher myself”: Involving Children as Co-Researchers in Analysis of Qualitative Paediatric Research

Luchtenberg ML, Maeckelberghe ELM, Verhagen AAE

University of Groningen, University Medical Center Groningen

Corresponding author:

Malou L. Luchtenberg

Postbus 30.001, 9700 RB Groningen

m.l.luchtenberg@umcg.nl, +31 6 25 64 96 59

Malou L. Luchtenberg

Department of Pediatrics, Beatrix Children’s Hospital

University of Groningen, University Medical Center Groningen, the Netherlands

Els L.M. Maeckelberghe

Institute for Medical Education

University of Groningen, University Medical Center Groningen, the Netherlands

A.A. Eduard Verhagen

Department of Pediatrics, Beatrix Children’s Hospital

University of Groningen, University Medical Center Groningen, the Netherlands

Word count: 3882 (excl. title page, abstract, references, tables and quotes)

Keywords: involvement, children, paediatrics, patient participation, biomedical research, qualitative research

For peer review only - http://bmjopen.bmj.com/site/about/guidelines.xhtml

ABSTRACT

Objectives
We aimed to test and reflect on a new approach of involving young children in analysing qualitative research.

Setting
Research meetings with children at their homes (phase one) or at their primary school (phase two). Phase one consisted of five one-on-one meetings between a child co-researcher and the adult researcher. Together, they watched an interview video to identify themes. The second phase consisted of two group meetings using video fragments from 23 interviews to further explore the identified themes.

Participants
We involved children of the public as co-researchers to analyse children's interviews about their experiences with taking part in medical research. Our co-researchers were between 10-14 years old. They provided feedback about their experiences.

Results
All child co-researchers identified themes that they thought were important. The extent to which they needed time and support in structuring varied. The child co-researchers led the discussion about the themes and made the final decision when disagreements occurred. The children rated the time investment as adequate and they valued being a co-researcher as interesting and fun, indicating that they learned new skills and gained knowledge. The experience also caused them to reflect on health and illness in their own lives. The process was relatively time intensive for the adult researchers, but resulted in a more critical assessment of their own work.

Conclusion
The two-phase approach is promising for actively involving young children in analysing qualitative data. We recommend using videos rather than transcripts to make it easier for children to understand the data and to empathize with the interviewees, and to limit the time investment.

STRENGTHS AND LIMITATIONS OF THIS STUDY

- This study describes a new two-phase approach to involving children in analysing qualitative interview data by means of individual and group meetings.
- This study explores the use of videos instead of transcripts to present the data to relatively young co-researchers.
- The study reflects on children's involvement as co-researchers both from the perspective of children themselves and from adult researchers.
- In this test phase, we limited the number of young co-researchers and selected interviews from a larger dataset, aiming to include as much variation as possible.

For peer review only - http://bmjopen.bmj.com/site/about/guidelines.xhtml

INTRODUCTION

Paediatric research is important to provide children with the best possible health care. Children are considered vulnerable, raising ethical concerns regarding their ability to give informed consent for research participation.[1] Yet, as stated in the United Nations Convention on the Rights of the Child, they have the right to express their views on everything that is affecting them, and should be provided the opportunity to be heard.[2] Therefore, in a larger as yet unpublished study, we collected experiences of young people on their participation in medical research. When analysing the interviews, we realized that to truly hear our interviewees' voices, the analysis should not be performed solely by adults.

In qualitative research, researchers need to be cautious about interpretation bias in the data analysis. Qualitative research includes a subjective component that makes reflexivity an important aspect of the research.[3] Since children have different life experiences and social situations, their interpretations of their peers' words may differ from adults' interpretations. Therefore, we wanted to involve children in our analysis, but there is little descriptive evidence on how children can be effectively involved in scientific data analysis.[4–11] Other challenges of patient and public involvement (PPI) [12] with children include a lack of funding and time, gatekeeping or power imbalances, and obtaining knowledge and training on how to involve children.[4,7,13–19] Best et al. recently introduced an interesting new method for involving a youth advisory PPI panel in qualitative data analysis: participatory theme elicitation.[20] However, a disadvantage of this method is that it involved data preselection by adults before the young researchers were involved.

We used elements of Best's promising method, but limited preselection. We also considered challenges such as time investment and training. We aimed to design a two-phase approach that would be both effective and efficient. In this paper we reflected on the process from the perspective of children and adults. A content analysis of differences between adult and child researcher interpretations will be published separately.

METHODS

Children were involved as co-researchers in analysis of interview data conducted in two phases, including five individual meetings in phase one, and two group meetings in phase two.

Recruitment and sampling

Phase 1

For peer review only - http://bmjopen.bmj.com/site/about/guidelines.xhtml
For peer review only

Potential co-researcher participants (9-18 years) were approached through national patient support organizations, primary schools, hospitals, social media, and word-of-mouth. No research experience was required. In this phase, five participants were involved. They attended primary or secondary school and were 10 to 14 years old. None of them had been involved as a co-researcher before.

Phase 2

Phase two participants were recruited in collaboration with an academic primary school in the Netherlands. In this school, both teachers and pupils are expected to have an academic mind-set aimed at research and analysis.[21] We invited one class (15 pupils) of the school's oldest students to participate. Ten pupils age 10 to 12 volunteered. One was unable to participate because of illness.

Informed consent

Phase 1

Potential participants received a verbal explanation and an information pack. They were asked to discuss the study with their parents, and to reply through mail or by telephone. Subsequently, we checked if they had understood the information and asked them to reconfirm their participation. Before the session started, the informed consent form was read and discussed with the researcher at the student's home. One parent was present, but did not interfere in the process. All participants, regardless of age, were asked to sign an informed consent form. In accordance with national health law regulations for minors, consent of one of the parents was obtained in addition to the child's consent.[22] Children were also asked to sign a confidentiality agreement regarding any personal information present in the data they analysed. At the end of the session, participants received a €10 voucher as compensation for their time and a certificate acknowledging their involvement as co-researchers.

Phase 2

Two researchers from the team (PPIA1 and PPIA2) visited the primary school to meet the pupils, teacher, and school head, and to introduce the research project. The primary school director gave her consent for the children to take part during school hours. The potential participants received an information leaflet similar to the phase one participants. They were asked to complete the consent form and the confidentiality agreement at home with a parent if they wanted to take part, and return the form to their schoolteacher. At the end of the group

For peer review only

meetings, the participants received a certificate, but were not provided with a voucher since they participated during school hours.

Data characteristics

The data from the original study, consisting of semi-structured, video-taped interviews with young people between 9 and 18 years old, about their experiences taking part in medical research were analysed by our co-researchers, the participants in this study. In addition, field notes were taken during the analyses by the adult researchers, and feedback was obtained from the participants before, during, and after taking part.

The co-researchers received a brief interactive introduction into paediatric research to stimulate collaboration between the researchers. We purposely did not give them extensive training, because we did not want them to become 'little adult researchers.' Also, extensive training would have been too time consuming for both the children and the researchers. The introduction and background information aimed to give the co-researchers an idea of what the data looked like and what it was about.

In the first phase, the co-researchers collaborated with the coordinating researcher (PPIA1) in a one-on-one session to find main themes in the interviews. They watched a video (25-45 minutes) of another young person and discussed emerging themes with PPIA1. This took place at the participants' home and lasted between two and three and a half hours. In the second phase, two identified themes were further explored in group meetings using video fragments of several interviews compiled into two short videos that each lasted five minutes. These took place at the children's primary school and lasted around two and a half hours.

The aim of the analysis process in both phases was to identify the main subjects present in the video through open, unstructured discussions. To facilitate interaction and discussion, both the participants and coordinating researcher could pause the video at will. They took notes of what they thought the interviewee found important. PPIA1 allowed moments of reflection on personal health and illness experiences, but she was alert regarding potential intertwining of researchers' personal experiences with interviewees' experience. Afterward, the participants developed strategies to make a mind map *("a type of diagram with lines and circles for organising information so that it is easier to use or remember")*[23, p1] in which they showed how they thought different themes were related to each other. The mind map was designed

For peer review only - http://bmjopen.bmj.com/site/about/guidelines.xhtml

using A3 paper, different size sticky notes, and different colour pens. All participants were free to use materials of their own choice. The researcher asked questions about the importance of certain subjects, the identification of an overlapping theme, and the reason why the participant(s) had chosen a certain subject. The participants led the discussion and made the final decision when there was disagreement about topics.

Data collection and analysis

All meetings were audio taped. The researchers made field notes of how participants fulfilled their role as co-researcher and the way they reflected on this process. Child co-researchers were asked to complete a feedback form about their experiences after the analysis took place (Table 1). In addition, the process was briefly orally evaluated, which was audio taped. All these experiences were collected and thematically analysed.

Table 1. Feedback questions that were asked of participants.

1. Did you understand beforehand what your role was in the project? (No / a little / yes)<br>2. How could we improve the information about working as a co-researcher?<br>3. What was it like for you to work as a co-researcher? (positive things, improvements points)<br>4. Have you learned anything from being a co-researcher? If so, what?<br>5. Would you like to be a co-researcher more often?	6. Would you advise other children to become co-researchers? Why?<br>7. How would you consider your time invested? (Too long, adequate, too short)<br>8. a. What did you think of the compensation voucher (€10)? (phase one)<br>b. What did you think about having this project during school hours? Why? (phase two)<br>9. Do you have advice on improving this evaluation form?

Patient and public involvement

This project explored a new approach of involving children in qualitative data analysis for research.

RESULTS

The results can be subdivided into adult researcher observations during the process itself, and experiences of all co-researchers. The experiences of adult and child co-researchers are summarized in Table 2.

For peer review only - http://bmjopen.bmj.com/site/about/guidelines.xhtml

For peer review only

Table 2. Summary of researchers' reflections and observations in involving children in interview analyses

	**Children**	**Adults**
Reasons to participate	Interesting and fun, something new	Better interpretation of children's voices, test method of involving children in analysis in efficient way, empowerment of children
Time investment	Acceptable	Significant but worthwhile
Reflection	On health and illness of interviewees and their own lives	On their own work
Lessons learned	New skills; taking notes, collaborating, critical thinking. Knowledge on diseases and experience in doing research.	Every child has something to add, as long as they are given the resources and structure. Children do have different interpretations than adults.
Reward (voucher)	Appreciated but not important for the younger children (age until 12 years primary school).	Important to acknowledge the work children do, to let them know they are part of the team. Reward according to wish of children.
Participation during school hours	Good because of the variation with schoolwork while keeping free time for themselves.	Convenient since children are gathered already which makes it easier to approach them for a group meeting.
Advise to peers	Do participate!	Do involve children!

Observations during the process

Co-researchers' understanding of informed consent procedure

The participants in the group meetings were very well able to recall the information they had received a month earlier; they especially remembered the concept of 'doing research about research.' Nonetheless, not all of them were aware of all the details that they and their parents had signed for. Even though the information was especially directed to the children, it was mostly read and signed by their parents at home, and not always discussed with the child. This was different from the co-researchers in the individual meetings. Both the researcher and the parents in the individual meetings were present at that moment and empowered the children to complete the form themselves, while the parents stayed available to help when necessary.

Co-researchers role development

For peer review only - http://bmjopen.bmj.com/site/about/guidelines.xhtml

During the analysis process, the videos were paused regularly by the participants or the adult researcher. During the first few minutes of the individual meetings this was mostly done by the researcher when she noticed that the participant needed more time to write notes, to discuss what the participant had written, and to ask how the participant experienced the process so far. When the adult researcher noticed it was going well, she took a step back to inspire confidence in the participants, and to stimulate them to take the lead. After a few minutes, the participants became more confident and a mutual collaboration arose; they took the lead in asking the researcher questions about her observations. Still, most participants continued to need some form of structuring support from the main researcher.

In the group meetings, role development was different. Given the logistics of being in a big group with one computer, the adult researcher put the video on pause every now and then. The participants were free to say 'stop' when they wanted the video to be paused. When the notes were transformed into the mind map, the facilitators noticed a clear distinction in participants' role preferences. Some found it hard to summarise their notes to be put on the mind maps, and brought up a solution by adding some steps in between. Others only needed a bit more time and space to find their own role.

Co-researchers' reflections on life and illness

The participants empathized with the young people who were sharing their experiences in the video. They wondered if the young people were still ill and asked if they were alive, hoping they were all right. They asked questions about the illnesses the young people had, and the consequences it had on their lives. They also shared experiences from their own lives, for example about relatives who had cancer or other illnesses. By communicating about illness, they better understood and identified with each other.

Co-researchers' interest in main interview study

Even though the participants were only involved in the analysis stage of the main interview study, they were well aware that this was part of a bigger study. They asked about the other participants and previous experiences with involving children in research analysis, and wondered whether the interviewees would see their mind maps that resulted from the analysis. Hearing that they were one of the first children that participated as co-researchers in this project made them feel special. They expressed the wish to get the project results and hoped we would do this project again. They also showed interest in why the adult researchers personally did this

For peer review only - http://bmjopen.bmj.com/site/about/guidelines.xhtml

research project and whether it was part of their university training, showing that the co-researchers had a broader interest than just fulfilling their role as co-researchers.

Co-researchers' experiences

Reasons to participate as co-researcher

The main reason participants became involved as co-researchers was that they thought it would be interesting and fun. Interest in research and medicine, earning money, and not having to work at school were less commonly reported as reasons to take part.

> *"I thought it could be fun and I had never done such a thing before, and I like to help people, and perhaps I want to become a doctor in the future so I thought it would be fantastic!" (PPI01)*

Co-researchers' understanding of the informed consent procedure

To improve the information, participants advised us to use fewer difficult words to explain it, to tell them specifically that they had to take notes of what they thought was important and that they had to create a mind map. More than half the participants did not have any suggestions for improving the information they received.

Experiences as co-researcher, including advise to other children

Participants liked being involved as co-researchers because it was fun and interesting; they were able to help other children; and they learned a lot themselves.

> *"It actually felt a bit like I was a researcher myself" (PPI09)*

The things they reported learning ranged from obtaining new knowledge about a certain health condition and experiencing what it is like to be ill, to learning how to do research, how to think critically, and how to take notes.

> *"You have to think carefully before you draw conclusions." (PPI07)*

> *"A bit about how ill children felt afterwards." (PPI14)*

They all reacted positively to the question whether they wanted to be a co-researcher more often.

For peer review only - http://bmjopen.bmj.com/site/about/guidelines.xhtml

"Yes it was fun, sociable and instructive" (PPI14)

Only one participant mentioned it would depend on whether he had the time to take part. This participant was the only one who attended secondary school, and consequently had more homework than other participants. All participants advised others to become a co-researcher, though one participant acknowledged that it might not suit everyone.

Reflection on time investment and reward

The meetings took much longer than we expected and described in the information sheets. We had estimated the meetings to last around two hours; instead the first meeting was three-and-a-half hours. This was then given special attention, and the next potential participants were informed and assured that they could pause or stop the meetings at any moment they wanted. The group meetings lasted as long as was planned, since we had to fit it within school hours. Most children said the time investment was adequate. One child from the group meetings mentioned that it would have been better if the meeting was shorter since some children in the group got distracted, which was also observed by the adult researchers. Another participant reported he had wanted it last longer because he really liked it and did not want to get back to his normal schoolwork.

The participants appreciated the certificates and vouchers very much. Two of the five phase one co-researchers specifically mentioned that for them it was not necessary to receive financial compensation.

Taking part during school hours

The phase two participants all reported that it was an interesting and nice alternative to the normal school tasks. They thought it was good to do the project during school time, because this way they would not miss out on any free time.

"It was fun because you didn't have to work, and if it hadn't been during school it was inconvenient" (PPI06)

Reflection of adult researchers

Lessons learned

For peer review only - http://bmjopen.bmj.com/site/about/guidelines.xhtml

The adult researchers learned a lot from involving young children in the analysis because the participants were very open and direct in their feedback. If they did not understand something, they directly expressed their struggles; for example when a question was not clearly formulated. They observed this in both the assertive and the shyer children. The adult researchers were challenged not to give answers themselves when the participants indicated that they did not know how to proceed at a certain point in their analysis. It helped the children when the adult researcher repeated the question they had asked or asked a similar question, without necessarily directing them, and acknowledged that they were doing the right thing. One of the adult researchers explained how she experienced the ability of children to interpret findings in many ways, and not to take one explanation for granted.

> *"I noticed that as an adult you tend to see things as obvious, for example which subtheme goes with a main theme, while children seem to have multiple other potential interpretations, and they are able to discuss those interpretations." (PPIA2)*

Time investment

In preparing the meetings, time was invested in recruitment, developing material to introduce and explain the procedure, and mostly in thinking about how to best involve children. The time investment in analysis with co-researchers was time-intensive but necessary for the comparison of adult and child analysis that will be reported elsewhere. We considered the time investment reasonable, given the reported empowerment of children, that they learned new skills, and that views of our data differed from ours, which is promising for better interpretation of our interviews. Apart from the time investment, there were no great project expenses. Materials were low cost, and there were minimal travel costs.

Personal reflections on children's involvement

Both adult researchers were positively surprised by the co-researchers' achievements, which made this project feasible and valuable for our data interpretation as well as personally rewarding.

> *"Experiencing how our co-researchers collaborated with us and each other in this research, the reflections they had and empathy they showed did not only empower them, it also made me feel empowered in the work we do." (PPIA1)*

For peer review only - http://bmjopen.bmj.com/site/about/guidelines.xhtml

DISCUSSION

Little evidence is available on how to involve children in research.[13] We described how relatively young children were involved in research interview analyses using a two-phase approach. Multiple strategies were used to avoid a tokenistic approach, to address challenges regarding time management, and to empower children in the process.

Two-phase approach

Involving relatively young children (aged 10-14) in our analysis was challenging. We tried to limit preselection of data by adults through one-on-one meetings in which entire interviews were analysed. Since these sessions were relatively long and intensive, it was an advantage that the adult researcher could focus solely on the individual co-researcher for further explanation and facilitation. The themes that were identified were further explored in the second phase through the group meetings. Working with multiple co-researchers in this phase improved the rigor of the qualitative analysis. In addition, there was an unexpected positive result for the co-researchers who were classmates. They strengthened their bond by sharing the research experience and reflecting on health and illness together. This two-phase approach made it possible to achieve our research goals and empower our co-researchers, while limiting the time invested for both adult and child researchers.

Use of videos in analysis

Our aim was active involvement of our co-researchers in interview analysis in an effective and efficient way. Data analysis in qualitative research is often a long and intensive process with large amounts of text. Locock et al. reported that reading the transcripts for young people in their study was difficult and inefficient. They concluded that it was more valuable to start a conversation and discuss the data rather than digging into the detailed transcripts.[11] Therefore, we decided to test other ways of involving young co-researchers in the analysis. Visuals such as photographs, drawings, or mapping methods are often used for collaboration with young children to collect data about children's views on, for example, the things they value in their lives.[24] Darbyshire et al. reported that using a variety of qualitative visual techniques was beneficial for getting children interested and engaged in research, and it provided a good way for children to express their views. There is a problem with using visuals as a participatory method rather than in analysis, as Darbyshire et al. pointed out: *"...having children take photographs and then having only adults 'interpret' (or possibly misinterpret) them is potentially an adultist approach to research on children that we sought to avoid"*.[25] Therefore, we used videos in the analysis stage to visualize the interview data that was to be

For peer review only - http://bmjopen.bmj.com/site/about/guidelines.xhtml

analysed. Our study confirmed the benefits expressed by Darbyshire et al. The co-researchers liked the creative process when they developed the mind map, and the videos helped them to understand and empathize with the interviewees. Using the videos instead of transcripts made it more time efficient, while preserving the effectiveness of a thematic analysis. Reflecting on this, we realized that using videos rather than transcripts has another benefit regarding rigor of the qualitative data analyses. Analysis of interview data is often assumed to start at the moment that the interview has been fully transcribed, but even when this is verbatim, including descriptions of vocal emotions such as laughter, there will always be a loss of key elements such as volume of voices and facial expressions. This could present interviewees' experiences in a more abstract way than the original data show.[26,27] In other words, by using videos, we might have started the analysis with a more authentic representation of our data.

Additional considerations and further research

Time investment for both adults and children is an important challenge for developing ways of involving children in research analysis. The method developed by Best et al. limited the analysis to two hours. Nevertheless, an additional time investment of four times 90-120 minutes was asked from participants for 'capacity building.' In these sessions, young people learned how to design and conduct a study, how to perform qualitative data analysis, and received an introduction into the subject of their data.[20] As described, we purposely did not train our co-researchers, both to avoid 'fitting' them into our own idea of what a qualitative researcher should be, and to limit time investment. Though we cannot make a comparison, the minimal training we provided to our co-researchers did not seem to have a negative impact on the result.

In addition to time investment, timing of research meetings should be considered. Many Young People's Advisory Groups (YPAGs) plan their meetings mostly during school holidays or on weekends.[28] INVOLVE identified parents and schools as a significant barriers to public involvement during school hours: *"…lack of schools' recognition of the value of their work sometimes acts as a barrier to them attending events which involve travel in school hours."*[15, p12] Nevertheless, we managed to set up a collaboration with a primary school for the second phase of our study, and planned the group meetings during school hours. The school director recognized the value of the research being carried out with children at school, perhaps because it is an academic primary school. The school director reported that the school is frequently approached for research projects throughout the school year, and it was therefore impossible not to do some gatekeeping. We were happy that the school valued our project and made an

For peer review only - http://bmjopen.bmj.com/site/about/guidelines.xhtml

exception for us. For our co-researchers in the group meetings, participation during school time was a great way to be involved, since they reported they had busy schedules in their free time. One of our co-researchers said that being busy with homework for secondary school might be a reason not to participate in similar project in the future.

We recently started testing our two-phase approach with young people age 16 to 18, who will use this project for a school assignment, thereby creating a win-win situation. If this proves beneficial, we will consider starting a long-term collaboration with both primary schools and secondary schools to optimize collaboration between researchers and children, which could be beneficial for both individual researchers and the schools involved. We expect that a comparison of findings between adults, children, and young people will bring interesting new insights from different life experiences and ages of the researchers involved.

Study limitations

To test this new approach, we started with a small group of young co-researchers. A consequence is that we had to make a preselection of interviews from a larger dataset. Within this selection we aimed to have as much variation as possible, but we had to consider pragmatic matters as well, such as the length of the interviews. In addition, in our method children were not involved in making choices about specific quotes used in the results sections.

CONCLUSION

Based on our study, we suggest that the two-phase approach provides an effective and efficient way to involve relatively young children in analysis of qualitative data by combining one-on-one meetings and group meetings. Presenting the interview data through videos rather than transcripts made it easier for children to understand the data, to empathize with the interviewees, and to limit time investment. The two-phase approach has the potential to prevent unrealistic interpretation of children's voices by adult researchers because it limits preselection of data by adults. Additional benefits are that children reflect on health and illness in their own lives, and they are empowered and engaged in medical research.

ETHICS APPROVAL

Both this study (M18.2334032 2018, July 24th) and our larger study (M16.192386 2016, May 10th) were concluded not to fall within the scope of the Medical Research Involving Human Subjects Act by the Medical Ethics Review Board of the University Medical Center Groningen.

ACKNOWLEDGEMENTS

For peer review only - http://bmjopen.bmj.com/site/about/guidelines.xhtml

We want to thank all participants in the original interview study, as well as our co-researcher participants from this project. Thanks to Laura Postma for co-facilitating the group meetings.

FUNDING STATEMENT

This PPI study itself had low costs. Data collection for the main interview study was funded by the University of Groningen as part of the MD/PhD program of Malou Luchtenberg. This funding body has no role in the design of the study and collection, analysis, or interpretation of data, or in writing the manuscript.

COMPETING INTERESTS STATEMENT

None declared.

DATA SHARING STATEMENT

The original dataset with audio- and videotaped interviews is not available for the public due to privacy concerns.

AUTHOR'S CONTRIBUTION

ML, EM and EV designed the original study. ML was responsible for the data collection in phase one, and the group meetings were led by ML and LP. ML made the first draft of the article, which was discussed regularly within the team and reviewed by EM and EV. All authors read and approved the final manuscript.

For peer review only

REFERENCES

1. World Medical Association. World Medical Association Declaration of Helsinki: ethical principles for medical research involving human subjects. *JAMA* 2013;310(20):2191–4. https://doi.org/10.1001/jama.2013.281053
2. U. N. General Assembly. Convention on the Rights of the Child, 20 November 1989. *Annu Rev Popul Law* 1989;16(September):501.
3. Hennink M, Hutter I, Bailey A. Qualitative Research Methods. London: Sage 2011.
4. Taylor RM, Whelan JS, Gibson F, et al. Involving young people in BRIGHTLIGHT from study inception to secondary data analysis: insights from 10 years of user involvement. *Res Involv Engagem* 2018;4(1):1–14. https://doi.org/10.1186/s40900-018-0135-x
5. Jamal F, Langford R, Daniels P, et al. Consulting with young people to inform systematic reviews: an example from a review on the effects of schools on health. *Heal Expect* 2015;18(6):3225–35. https://doi.org/10.1111/hex.12312
6. Coad J, Evans R. Reflections on practical approaches to involving children and young people in the data analysis process. *Child Soc* 2008 Jan;22(1):41–52. https://doi.org/10.1111/j.1099-0860.2006.00062.x
7. Clavering EK, McLaughlin J. Children's participation in health research: from objects to agents? *Child Care Health Dev* 2010;36(5):603–11. https://doi.org/10.1111/j.1365-2214.2010.01094.x
8. Brady L-M, Preston J. Evaluating the extent and impact of young people's involvement in National Institute for Health Research (NIHR) studies: an assessment of feasibility. 2017 April. http://www.invo.org.uk/wp-content/uploads/2017/06/Impact-of-CYPs-involvement_JLI__GenR.pdf (accessed 01-07-2019)
9. Lundy L, McEvoy L, Byrne B. Working with young children as co-researchers: an approach informed by the United Nations Convention on the Rights of the Child. *Early Educ Dev* 2011;22(5):714–36. https://doi.org/10.1080/10409289.2011.596463
10. Holland S, Renold E, Ross NJ, et al. Power, agency and participatory agendas: a critical exploration of young people's engagement in participative qualitative research. *Childhood* 2010;17(3):360–75. https://doi.org/10.1177/0907568210369310
11. Locock L, Kirkpatrick S, Brading L, et al. Involving service users in the qualitative analysis of patient narratives to support healthcare quality improvement. *Res Involv Engagem* 2019;5(1):1–11. https://doi.org/10.1186/s40900-018-0133-z
12. INVOLVE. What is public involvement in research? *NHS Natl Inst Heal Res* 2015. http://www.invo.org.uk/find-out-more/what-is-public-involvement-in-research-2/ (accessed 10-07-2019)
13. Bird D, Culley L, Lakhanpaul M. Why collaborate with children in health research: an analysis of the risks and benefits of collaboration with children. *Arch Dis Child - Educ Pract Ed* 2013;98(2):42–8. https://doi.org/10.1136/archdischild-2012-303470
14. Preston J, Stones SR, Davies H, et al. How to involve children and young people in what is, after all, their research. *Arch Dis Child* 2019;104(5):494–500. https://doi.org/10.1136/archdischild-2018-315118
15. Sonpal K, Walker E, Swallow V, et al. Report on involving children and young people in research. Southampton: INVOLVE. 2019. https://www.invo.org.uk/wp-content/uploads/2019/06/77620-YPv3-Booklet-WEB.pdf (accessed 01-07-2019)
16. Blackburn S, McLachlan S, Jowett S, et al. The extent, quality and impact of patient and public involvement in primary care research: a mixed methods study. *Res Involv Engagem* 2018;4(1):1–18. https://doi.org/10.1186/s40900-018-0100-8

For peer review only - http://bmjopen.bmj.com/site/about/guidelines.xhtml

17. Domecq JP, Prutsky G, Elraiyah T, et al. Patient engagement in research: a systematic review. *BMC Heal Serv Res* 2014;14(89):1–9. https://doi.org/10.1186/1472-6963-14-89
18. Kellett M, Forrest R, Dent N, et al. “Just teach us the skills please, we’ll do the rest”: Empowering ten-year-olds as active researchers. *Child Soc* 2004;18(5):329–43. https://doi.org/10.1002/chi.807
19. Chappell P, Rule P, Dlamini M, et al. Troubling power dynamics: youth with disabilities as co-researchers in sexuality research in South Africa. *Childhood* 2014;21(3):385–99. https://doi.org/10.1177/0907568214525427
20. Best P, Badham J, Corepal R, et al. Network methods to support user involvement in qualitative data analyses: an introduction to Participatory Theme Elicitation. *Trials* 2017;18(1):1–14. https://doi.org/10.1186/s13063-017-2289-5
21. Brederoschool. Academische Basisschool 2019. Groningen, the Netherlands. https://brederoschool.openbaaronderwijsgroningen.nl/academischebasisschool/index.html (accessed 09-11-2018)
22. Dutch Medical Research Involving Human Subjects Act (WMO), 26 February 1998. https://wetten.overheid.nl/BWBR0009408/2019-04-02 (accessed 09-04-2019)
23. Cambridge University Press. Meaning of mind map in English. Cambridge Dictionary. 2019. https://dictionary.cambridge.org/dictionary/english/mind-map (accessed 19-09-2019)
24. Coad J. Using art-based techniques in engaging children and young people in health care consultations and/or research. *J Res Nurs* 2007;12(5):487–97. https://doi.org/10.1177/1744987107081250
25. Darbyshire P, Macdougall C, Schiller W. Multiple methods in qualitative research with children: more insight or just more? *Qual Res* 2005;5(4):417–436. https://doi.org/10.1177/1468794105056921
26. Hewitt J. Ethical components of researcher-researched relationships in qualitative interviewing. *Qual Health Res* 2007;17(8):1149–59. https://doi.org/10.1177/1049732307308305
27. Alldred P, Gillies V. Eliciting research accounts: re/producing modern subjects. Ethics in Qualitative Research. London: Sage 2002:146–65.
28. Gaillard S, Malik S, Preston J, et al. Involving children and young people in clinical research through the forum of a European Young Persons’ Advisory Group: needs and challenges. *Fundam Clin Pharmacol* 2018 Aug;32(4):357–62. https://doi.org/10.1111/fcp.12360

For peer review only - http://bmjopen.bmj.com/site/about/guidelines.xhtml

CERTIFICATE OF ENGLISH EDITING

This document certifies that the paper listed below has been edited to ensure that the language is clear and free of errors. The logical presentation of ideas and the structure of the paper were also checked during the editing process. The edit was performed by professional editors at Editage, a division of Cactus Communications. The intent of the author's message was not altered in any way during the editing process. The quality of the edit has been guaranteed, with the assumption that our suggested changes have been accepted and have not been further altered without the knowledge of our editors.

Title Of The Paper

"I actually felt like I was a researcher myself": Involving Children as Co-Researchers in Analysis of Qualitative Paediatric Research

Authors

Malou L. Luchtenberg

Job Code

ALCHT_1

editage by CACTUS

Signature

Vikas Narang

Vikas Narang,
Senior Vice President,
Operations-Author Services, Editage

Date of Issue
September 16, 2019

Editage, a brand of Cactus Communications, offers professional English language editing and publication support services to authors engaged in over 500 areas of research. Through its community of experienced editors, which includes doctors, engineers, published scientists, and researchers with peer review experience, Editage has successfully helped authors get published in internationally reputed journals. Authors who work with Editage are guaranteed excellent language quality and timely delivery.

INFORMATION SECURITY MANAGEMENT SYSTEM DNV·GL ISO/IEC 27001

CACTUS

Contact Editage

Worldwide	Japan	Korea	China	Brazil	Taiwan
request@editage.com	submissions@editage.com	submit-korea@editage.com	fabiao@editage.cn	contato@editage.com	submitjobs@editage.com
+1 877-334-8243	+81 03-6868-3348	1544-9241	400-005-6055	0800-892-20-97	02 2657 0306
www.editage.com	www.editage.jp	www.editage.co.kr	www.editage.cn	www.editage.com.br	www.editage.com.tw

BMJ Open

"I actually felt like I was a researcher myself": Involving Children as Co-Researchers in Analysis of Qualitative Paediatric Research

Journal:	*BMJ Open*
Manuscript ID	bmjopen-2019-034433.R1
Article Type:	Original research
Date Submitted by the Author:	22-Jan-2020
Complete List of Authors:	Luchtenberg, Malou; University of Groningen; University Medical Center Groningen, Department of Pediatrics<br>Maeckelberghe, Els; University of Groningen, Institute for Medical Education; University Medical Center Groningen<br>Verhagen, Eduard; University of Groningen; University Medical Center Groningen , Department of Pediatrics
**Primary Subject Heading**:	Paediatrics
Secondary Subject Heading:	Ethics, Qualitative research, Research methods
Keywords:	Involvement, Children, PAEDIATRICS, Patient Participation, Biomedical Research, QUALITATIVE RESEARCH

SCHOLARONE™
Manuscripts

I, the Submitting Author has the right to grant and does grant on behalf of all authors of the Work (as defined in the below author licence), an exclusive licence and/or a non-exclusive licence for contributions from authors who are: i) UK Crown employees; ii) where BMJ has agreed a CC-BY licence shall apply, and/or iii) in accordance with the terms applicable for US Federal Government officers or employees acting as part of their official duties; on a worldwide, perpetual, irrevocable, royalty-free basis to BMJ Publishing Group Ltd ("BMJ") its licensees and where the relevant Journal is co-owned by BMJ to the co-owners of the Journal, to publish the Work in this journal and any other BMJ products and to exploit all rights, as set out in our licence.

The Submitting Author accepts and understands that any supply made under these terms is made by BMJ to the Submitting Author unless you are acting as an employee on behalf of your employer or a postgraduate student of an affiliated institution which is paying any applicable article publishing charge ("APC") for Open Access articles. Where the Submitting Author wishes to make the Work available on an Open Access basis (and intends to pay the relevant APC), the terms of reuse of such Open Access shall be governed by a Creative Commons licence – details of these licences and which Creative Commons licence will apply to this Work are set out in our licence referred to above.

Other than as permitted in any relevant BMJ Author's Self Archiving Policies, I confirm this Work has not been accepted for publication elsewhere, is not being considered for publication elsewhere and does not duplicate material already published. I confirm all authors consent to publication of this Work and authorise the granting of this licence.

For peer review only

"I actually felt like I was a researcher myself": Involving Children as Co-Researchers in Analysis of Qualitative Paediatric Research

Malou Luchtenber, Els Maeckelberghe, Eduard Verhagen

University of Groningen, University Medical Center Groningen

Corresponding author:

Malou L. Luchtenberg

Postbus 30.001, 9700 RB Groningen

m.l.luchtenberg@umcg.nl, +31 6 25 64 96 59

Malou L. Luchtenberg

Department of Pediatrics, Beatrix Children's Hospital

University of Groningen, University Medical Center Groningen, the Netherlands

Els L.M. Maeckelberghe

Institute for Medical Education

University of Groningen, University Medical Center Groningen, the Netherlands

A.A. Eduard Verhagen

Department of Pediatrics, Beatrix Children's Hospital

University of Groningen, University Medical Center Groningen, the Netherlands

Word count: 3882 (excl. title page, abstract, references, tables and quotes)

Keywords: involvement, children, paediatrics, patient participation, biomedical research, qualitative research

For peer review only - http://bmjopen.bmj.com/site/about/guidelines.xhtml

ABSTRACT

Objectives
We aimed to test and reflect on a new approach of involving young children in analysing qualitative research.

Setting
Single-centre study in the Netherlands including research meetings with children at their homes (phase one) or at their school (phase two). Phase one consisted of five one-on-one meetings between a child co-researcher and the adult researcher to identify themes from a video interview. Phase two consisted of two group meetings to further explore the identified themes using fragments from 23 interviews.

Participants
We involved children (aged 10-14) of the public as co-researchers to analyse children's interviews about their experiences with taking part in medical research. They provided feedback about their experiences.

Results
All child co-researchers identified themes that they thought were important. The extent to which they needed time and support in structuring varied. The children themselves rated the time investment as adequate and they valued being a co-researcher as interesting and fun, indicating that they learned new skills and gained knowledge. The experience also caused them to reflect on health and illness in their own lives. The process was relatively time intensive for the adult researchers, but resulted in a more critical assessment of their own work.

Conclusion
The two-phase approach is promising for actively involving young children in analysing qualitative data. It has the potential to prevent unrealistic interpretation of children's voices by adult researchers because it limits preselection of data by adults. We recommend using videos rather than transcripts to make it easier for children to understand the data and to empathize with the interviewees, and to limit the time investment.

STRENGTHS AND LIMITATIONS OF THIS STUDY

- This study describes a new two-phase approach to involving children in analysing qualitative interview data by means of individual and group meetings.
- This study explores the use of videos instead of transcripts to present the data to relatively young co-researchers.
- The study reflects on children's involvement as co-researchers both from the perspective of children themselves and from adult researchers.
- In this test phase, we limited the number of young co-researchers and selected interviews from a larger dataset, aiming to include as much variation as possible.

For peer review only

For peer review only - http://bmjopen.bmj.com/site/about/guidelines.xhtml

INTRODUCTION

Paediatric research is important to provide children with the best possible evidence based health care. Children are considered vulnerable, raising ethical concerns regarding their ability to give informed consent for research participation.[1] This results in reluctance to invite children Therefore, in a larger as yet unpublished study, we collected experiences of young people on their participation in medical research to provide recommendations for improvement of children's participation in research. In order to truly hear the interviewees' voices, we must take them seriously, and children should be involved in the analysis.

In qualitative studies, researchers need to be cautious about interpretation bias in the data analysis. Qualitative research includes a subjective component that makes reflexivity important.[2] Since children have different life experiences and social situations, their interpretations of their peers' words may differ from adults' interpretations. Therefore, we wanted to involve children in our analysis to strengthen our analysis, but there is little descriptive evidence on how children can be effectively involved in scientific data analysis.[3–10] Other challenges of patient and public involvement (PPI) [11] with children include a lack of funding and time, gatekeeping or power imbalances, and obtaining knowledge and training on how to involve children.[3,6,12–18] Best et al. recently introduced an interesting new method for involving a youth advisory PPI panel in qualitative data analysis: participatory theme elicitation.[19] However, a disadvantage of this method is that it involved data preselection by adults before the young researchers were involved.

We used elements of Best's promising method, but limited preselection. We also considered challenges such as time investment and training. Therefore, we designed a two-phase approach that would be both effective, i.e. hearing children's true voices, and efficient, i.e. limiting time investment. In this paper we reflect on how we involved children in data analysis, and what we learned from this process from the perspective of children and adults. Measuring the impact of a PPI process in terms of the content is another great challenge in this field of research.[20] This will be evaluated and published separately.

METHODS

This is a single-centre study conducted at the University Medical Center Groningen, the Netherlands. Children were involved as co-researchers in analysis of interview data conducted in two phases, including five individual meetings in phase one, and two group meetings in phase

For peer review only - http://bmjopen.bmj.com/site/about/guidelines.xhtml

two. This was an exploratory study.

Recruitment and sampling

Phase 1

Potential co-researcher participants were approached through national patient support organizations, primary schools, hospitals, social media, and word-of-mouth. No research experience was required. Sampling was based on age (9-18 years) and language (fluency in Dutch). Though recruitment was challenging, we reached our goal of five participants in this first phase.

Phase 2

Phase two participants were recruited in collaboration with a local academic primary school in Groningen, the Netherlands, where teachers and pupils are expected to have an academic mind-set aimed at research and analysis.[21] We invited one class (15 pupils) of the school's oldest students to participate. Ten pupils volunteered, one was unable to participate because of illness.

Informed consent

Phase 1

Potential participants received verbal and written information. They were asked to discuss the study with their parents, and to reply through mail or by telephone. Before the session started, the informed consent form was read and discussed with the researcher at the child's home. One parent was present, but did not interfere in the process. All children (regardless of age) were asked to sign informed consent to acknowledge their unique and equally valued contribution. In accordance with the Dutch Medical Research Involving Human Subjects Act (WMO), parental consent was obtained in addition to the child's consent.[22] Children were also asked to sign a confidentiality agreement regarding any personal information present in the data they analysed. At the end of the session, participants received a €10 voucher as compensation for their time and a certificate acknowledging their contribution as co-researchers.

Phase 2

Two adult researchers visited the primary school to meet the pupils, teacher, and headmaster, and to introduce the research project. The headmaster gave consent for the children to participate during school hours. Potential participants received an information leaflet similar to the phase one participants. They were asked to complete the consent form and the

For peer review only - http://bmjopen.bmj.com/site/about/guidelines.xhtml

confidentiality agreement at home with a parent, and return the form to their schoolteacher. At the end of the group meetings, the participants received a certificate, but were not provided with a voucher since they participated during school hours.

Data characteristics

The data from the original study were analysed by our co-researchers. In addition, field notes were taken during the analyses by the adult researchers, and feedback was obtained from the participants before, during, and after participating. Figure 1 provides an overview of the data presented in this study. Study 1 represents the original qualitative interview study. Study 2 refers to the two-phase project in which children collaborated with adult researchers as co-researchers in analysing the data of study 1. Study 3 is the study reported on in this paper. Table 1 provides more information on the data collected for the original study.

Table 1. Details of data of the larger interview study on children's experiences in research

**Study characteristics**	
**Aim**	To explore children's experiences in medical research to provide recommendation from their own perspectives on how to improve their involvement in research.
**Setting and research team**	Single-centre study performed by research team at the University Medical Center Groningen, the Netherlands. The research team consisted of an ethicist (EM), paediatrician (EV), and MD/PhD student (ML). All members are trained and/or have experience in qualitative research.
**Recruitment and sampling**	Recruitment through health providers from multiple hospitals, national patient support groups, social media, and word-of-mouth. Purposive maximum variation sample: children 9-18 years old who were invited to participate in different types of medical research in the Netherlands (and took part or declined to take part), both patients and healthy volunteers. Participants had no prior relationship with the research team.
**Informed consent**	Informed consent from a parent and child, or child only (from 16 years on) according to Dutch law regulations.
**Data collection**	23 Semi-structured in-depth interviews (lasting between 30-100 min) with children about their experiences with taking part in research, including advices for improvement of their involvement in informed consent procedure and the research itself. A topic guide was developed based on a previous study in the UK. Interviews performed by ML took place at children's homes and were audio or video taped, transcribed verbatim, and returned to the participants (no comments from participants were received). Data collection lasted until data saturation was reached.
**Ethics approval**	This study (M16.192386 2016, May 10th) was concluded not to fall within the scope of the Medical Research Involving Human Subjects Act (WMO) by the Medical Ethics Review Board of the University Medical Center Groningen.

The co-researchers received a brief interactive introduction into paediatric research and study 1. They were not given extensive training, to avoid them to become 'little adult researchers'. Furthermore, extensive training would have been time consuming for both the children and the researchers.

For peer review only - http://bmjopen.bmj.com/site/about/guidelines.xhtml

In phase 1, the co-researchers collaborated with the coordinating researcher (PPIA1 – PPI adult researcher 1) in a one-on-one session to find main themes in the interviews of study 1. They watched a video (25-45 minutes) of another young person and discussed emerging themes. This took place at the participants' home and lasted between two and three and a half hours. In the second phase, two identified themes were further explored in group meetings using video fragments of several interviews compiled into two short videos that each lasted five minutes. These group meetings took place at one local primary school and lasted around two and a half hours.

The aim of the analysis process in both phases was to identify the main topics in the video through open, unstructured discussions. To facilitate interaction and discussion, both participants and coordinating researcher could pause the video at will. They took notes of what they thought the interviewee found important. PPIA1 allowed moments of personal reflection, but she was alert regarding potential intertwining of researchers' personal experiences with interviewees' experiences. Afterward, the participants created a mind map *("a type of diagram with lines and circles for organising information so that it is easier to use or remember")*[23, p1] depicting their interpretation of the interrelatedness of different themes. The mind map was designed using A3 paper, different sizes of sticky notes, and colour pens. All participants were free to use materials of their own choice. The researcher asked questions about the importance of certain subjects, the identification of an overlapping theme, and the reason why the participant(s) had chosen a certain subject. The participants led the discussion and made the final decision when there was disagreement about topics.

Data collection and analysis

Before the project started, child co-researchers were asked why they wanted to take part. The adult researchers made field notes of how participants fulfilled their role as co-researcher, and how the participants reflected on this process. Child co-researchers completed a feedback form after the analysis (Table 2). In addition, the process was briefly orally evaluated. All meetings were audio taped.

The analysis was done by PPIA1 (ML) using a framework approach,[24] and discussed with EM (individual meetings) and PPIA2 (group meetings). Some themes, such as time investment, were identified in advance from literature, others were derived from the data. Regular updates

For peer review only

were given to the research team, and any disagreements were discussed. As this exploratory study had a relatively small sample size, we did not aim for data saturation.

Table 2. Feedback form

1. Did you understand beforehand what your role was in the project? (No / a little / yes)
2. How could we improve the information about working as a co-researcher?
3. What was it like for you to work as a co-researcher?
   a. positive things
   b. improvements points
4. Have you learned anything from being a co-researcher? If so, what?
5. Would you like to be a co-researcher more often?
6. Would you advise other children to become co-researchers? Why?
7. How would you consider your time invested? (Too long, adequate, too short)
8. a. What did you think of the compensation voucher (€10)? (phase one)
   b. What did you think about having this project during school hours? Why? (phase two)
9. Do you have advice on improving this evaluation form?

For peer review only

Patient and public involvement

This project explored a new approach of involving children in qualitative data analysis for research.

RESULTS

Participant characteristics

In total, fourteen children, eight girls and six boys, participated as co-researchers in this study. Two participants had experience being a chronic patient. None of them had been a co-researcher before. Table 3 and 4 show characteristics of child and adult participants, respectively.

Table 3. Child participants characteristics

**Characteristics child co-researchers**	**N* (%)****
Sex	
Girl	8 (57)
Boy	6 (43)
Age	
10 years	1 (7)
11 years	10 (71)
12 years	2 (14)
13 years	0 (0)
14 years	1 (7)
School attending	
Primary School	13 (93)
Secondary School	1 (7)
Hospital/Disease experience (lived experience)	
Currently having a disease	2 (14)
Hospitalization or small surgery in the past	6 (43)
Having family members that have a disease	2 (14)
None	4 (29)

For peer review only - http://bmjopen.bmj.com/site/about/guidelines.xhtml

Research experience as participant	
Yes	2 (14)
No	12 (86)
Experience as co-researcher	
Yes	0 (0)
No	14 (100)

* Number of child participants; total N = 14
** Rounded to nearest whole number

Table 4. Adult participants characteristics

Participant	Age	Sex	Experience illness /hospital	Research experience (participation in research)	Research experience (performing research)
PPIA1	27	F	As a medical student. Graduated as medical doctor (Aug 2019).	Yes, participating in two big cohort studies for several years now.	Training and experience in qualitative research during PhD.
PPIA2	23	F	As a medical student. Started internships half a year ago.	No experience in research participation.	Trained in qualitative research as a former psychology student.

Reflection and evaluation of the involvement process

The results can be divided into 5 themes: (1) understanding of study procedures, (2) empowerment, (3) reflection on health and illness, (4) interest in the bigger picture, and (5) reflection on time investment. The results of the feedback form are summarized in Table 5.

Table 5. Summary of feedback

Question	Summary of answers (number of responses)*
1. Did you understand beforehand what your role was in the project? (No / a little / yes)	No (0)<br>A little (12)<br>Yes (2)
2. How could we improve the information about working as a co-researcher?	Don't know (4)<br>Everything was clear (5)<br>Using less difficult words (4)<br>Saying that we are going to take notes and create a mind map (1)
3. What was it like for you to work as a co-researcher?<br>a. positive things<br>b. improvements points	a. Fun (14), interesting (4), helping other children (1), you learn yourself (1), time investment was okay (2), receiving a certificate (1)<br><br>b. no improvement points (12), shorter interviews (1), working an entire school day (instead of a half one) (1)
4. Have you learned anything from being a co-researcher? If so, what?	Taking notes (2)<br>Critical thinking and listening (4)<br>About a medical condition (2)<br>About doing research (2)<br>About how children think and feel about research (2)<br>That children think different from adults (1)<br>That it is fun and you learn a lot (1)<br>Not really (1)

For peer review only - http://bmjopen.bmj.com/site/about/guidelines.xhtml

5. Would you like to be a co-researcher more often?	Yes, because it's fun (12)<br>Yes, because it's interesting (5)<br>Yes, because I like to help people (1)<br>Yes, I know what to expect now (1)<br>Yes, if it doesn't hurt (1)<br>It is fun but depends on the time I have (1)
6. Would you advise other children to become co-researchers? Why?	Yes, because it's (super) fun (9)<br>Yes, because it's interesting/you learn something from it (7)<br>Yes, because you receive a voucher (1)<br>Yes, because you can help other people (2)<br>Yes, because you get candy (1)<br>Yes, but it depends if it fits them (1)
7. How would you consider your time invested? (Too long, adequate, too short)	Too long (1)<br>Adequate (12)<br>Too short (1; in between too short and adequate)
8. a. What did you think of the compensation voucher (€10)? (phase one)<br>b. What did you think about having this project during school hours? Why? (phase two)	a. Fun/good (5) …but not necessary (2), creative (1)<br><br>b. Fun/good, …because you didn't have to work on school things (6) …because you don't miss free time (4), don't mind (1)
9. Do you have advice on improving this evaluation form?	Adding a question about the overall experience (1)<br>No (13)

* Some participants gave multiple answers

1. Understanding of study procedures

The participants in the group meetings were able to recall the main idea of "*doing research about research*", which they expressed when they were asked about their expectations of the session. Nonetheless, not everybody remembered the details, such as whether pictures would be taken. Even though the information was especially directed to the children, the participants recalled that it was mostly read and signed by their parents at home, and not always discussed with them. This was different from the co-researchers in the individual meetings where both the researcher and the parents were present and empowered the children to complete the form themselves. Parents stayed available to help when necessary.

Most children mentioned that they had a generalized idea about their role in the project. Their specific role was clarified during the actual project. One of them explained:

> *"I already understood it but once you are doing it you understand it [better]."* (girl, 11 years old, individual meeting)

2. Empowerment

For peer review only - http://bmjopen.bmj.com/site/about/guidelines.xhtml

As a result of being involved as co-researchers, children gained knowledge, they learned new skills, and they became more confident in fulfilling their role during the analysis process. This was mostly due to having fun, experiencing something new, and being able to contribute to research or helping others. Children felt empowered by working as a co-researcher, as one of them explained:

> *"It actually felt a bit like I was a researcher myself"* (girl, 11 years old, group meeting)

They reported obtaining new knowledge about a certain health condition and about experiencing what it is like to be ill. They learned how to do research, how to think critically, and how to take notes. Some representative answers were:

> *"You have to think carefully before you draw conclusions."* (boy, 11 years old, group meeting)

> *"A bit about how ill children felt afterwards [participating in research]."* (girl, 11 years old, group meeting)

All participants were positive about the idea of being a co-researcher more often, mostly for similar reasons as why they wanted to take part in the first place:

> *"Yes it was fun, sociable and instructive"* (girl, 11 years old, group meeting)

Only one participant mentioned it would depend on whether he had the time to take part. He explained this was due to his homework and sports activities in his free time. This participant was the only one who attended secondary school. All participants advised others to become a co-researcher, though one participant acknowledged that it might not suit everyone. She mentioned that some children might not like or have the skills to do such work.

During the analysis process in phase one, the co-researchers noticeably became more confident as the time proceeded, and the adult researcher retreated to inspire confidence, and stimulate them to take the lead. Eventually, most participants took the lead, and, for example, also asked

For peer review only - http://bmjopen.bmj.com/site/about/guidelines.xhtml

the researcher questions about her observations, instead of the other way around. Still, most participants continued to need some form of structuring support from the main researcher.

In the group meetings, the support needed from the adult researcher was a bit different. The co-researchers needed more structuring due to group dynamics. Chaos emerged when multiple co-researchers started to talk at the same time, and shyer children tended to not be heard. Interestingly, the two groups chose different ways of transforming their notes into the mind map. In one group, the adult researcher noticed a clear distinction in participants' role preferences, and the co-researcher divided roles together. Some wanted an executive role, such as writing down themes on the 'sticky notes', while others preferred a position in which they could just express their ideas and have a more coordinating role. Some found it hard to summarise their notes, and brought up a solution by adding some steps, such as underlining important notes:

> *"We could also just first underline what we think is important."* (girl, 11 years old, group meeting)

The co-researchers in the other group, however, decided together that they all wanted to write down their own notes on 'sticky notes', and have them all on the mind map. The outcome was a mind map that not only showed different topics, it also gave insight into how important the individuals thought a certain topic by the amount of 'sticky notes' of the same topic. Others only needed a bit more time and space to find their own role.

The adult researchers were challenged not to give answers themselves when the participants indicated that they did not know how to proceed at a certain point in their analysis. Children felt reinforced when the adult researcher repeated or rephrased their question, acknowledging that they were doing the right thing. Both adult researchers were positively surprised by the co-researcher's achievements, which made this project feasible and likely to be valuable for the data interpretation as well as personally rewarding.

> *"Experiencing how our co-researchers collaborated with us and each other in this research, the reflections they had and empathy they showed did not only empower them, it also made me feel empowered in the work we do." (adult researcher, female, 27 years old)*

For peer review only - http://bmjopen.bmj.com/site/about/guidelines.xhtml

They learned a lot from involving children in the analysis because the participants were very open and direct in their feedback. If they did not understand something, they directly expressed their struggles, for example when a question was not clearly formulated, or when they did not understand medical jargon such as ‘treatment protocol’.

Whereas the adult researchers tend to generalize findings when analysing the data, the child-co-researchers stuck more to the original data. One topic was, for example, advices to researchers for improvement of young people’s experiences during participation in research. The interviewees mentioned things like making a hospital visit more fun or enjoyable, and gave concrete examples how to make it more pleasant. All researchers (adults and children) started from the original data, but there seemed to be a difference in the analysis. In children’s analysis concrete examples of those advices remained, thereby putting emphasis on them, while adults generalised them into ‘things to brighten up the visits’. The child co-researchers brought the adults back to the details, which seem to be important to children.

3. Reflection on health and illness

The participants empathized with the young people who were sharing their experiences in the video. They wondered if the young people were still ill and asked if they were alive, hoping they were all right. The following is a representative example:

> *“Nobody of these children [in the video] is deadly ill, right?”* (girl, 11 years old, group meeting)

The adult researcher explained the different conditions the interviewees had, and mentioned that some of them had been critically ill (e.g. having leukemia), but that they were stable when they were interviewed. The conversation then continued as followed:

> *“But it is going better now?”* (girl, 11 years old, group meeting)
> *“Yes, do you think that is important?”* (adult researcher, female, 27 years old)
> *“Yes, I do, … if you see someone like that [at the video].”* (girl, 11 years old, group meeting) *“...that would be a sad summary [otherwise].”* (boy, 11 years old, group meeting)

For peer review only - http://bmjopen.bmj.com/site/about/guidelines.xhtml

The co-researchers asked questions about the illnesses, and its consequences on the lives of the children, for example for two siblings with a hereditary condition. They also shared experiences from their own lives, for example about relatives who had cancer.

4. Interest in the bigger picture

Even though the participants were only involved in the analysis stage of the main interview study, they were well aware that this was part of a bigger study. They asked about the other participants and previous experiences with involving children in research analysis, and wondered whether the interviewees would see their mind maps that resulted from the analysis. Some of the questions they asked were:

> *"Have you been at children's homes?" "Are you doing this [project] on other schools too?" "How many times have you done this?"* (multiple co-researchers at the focus groups, boy/girl, 11/12 years old, who were not clearly identifiable from the audio)

Hearing that they were the first group of children that participated as co-researchers in this project made them feel special. They expressed the wish to receive the final results and hoped we would do this project again. The co-researchers had a broader interest than just fulfilling their role as co-researchers, and asked why the adult researchers personally did this research project and whether it was part of their university training. They also acknowledged and liked that they were able to help the adult researchers with their research:

> *"It is off course good for you [adult researchers] that we participate so that you can continue doing research about research of the research."* (girl, 11 years old, group meeting)

5. Reflection on time investment

Most children said the time investment was adequate. One child from the group meetings mentioned that it would have been better if the meeting was shorter since some children in the group got distracted:

> "*Because at the end we were chatting a bit, [we got] distracted." [boy, 11 years old, group meeting]*

For peer review only - http://bmjopen.bmj.com/site/about/guidelines.xhtml

This was also observed by the adult researchers. Another participant, however, reported he had wanted it to last longer because he really liked it and did not want to get back to his normal schoolwork. The phase two participants all reported that it was an interesting and nice alternative to the normal school tasks. They thought it was good to do the project during school time, because this way they would not miss out on any free time. One of the participants explained this:

> *"It was fun because you didn't have to work, and if it hadn't been during school it was inconvenient" (girl, 11 years old)*

From the adult perspective, time was invested in recruitment, developing material to introduce and explain the procedure, and mostly in thinking about how to best involve children. The time investment in analysis with co-researchers was time-intensive, and lasted longer than we had expected. However, the time investment was considered reasonable, given the reported empowerment of children, that they learned new skills, and that views of our data seemed to differ from ours, which is promising for better interpretation of our interviews. Materials were low cost, and there were minimal travel costs.

DISCUSSION

Little evidence is available on how to involve children in research.[12] We described how relatively young children were involved in research interview analysis using a two-phase approach. Multiple strategies were used to avoid a tokenistic approach, to address challenges regarding time management, and to empower children in the process.

Two-phase approach

Involving relatively young children (aged 10-14) in our analysis was challenging. We tried to limit preselection of data by adults through one-on-one meetings in which entire interviews were analysed. This might add value to Best's promising 'participatory theme elicitation' method.[19] Since these sessions were relatively long and intensive, it was an advantage that the adult researcher could focus solely on the individual co-researcher for further explanation and facilitation. We believe it also helped that the individual meetings took place at children's homes, as this is a safe and familiar environment for them. This confirms findings from Dovey-Pearce et al who highlighted the importance of having face-to-face meetings to build on relationships.[20] The themes identified were further explored in the second phase through the

For peer review only - http://bmjopen.bmj.com/site/about/guidelines.xhtml

group meetings. Working with multiple co-researchers in this phase improved the rigor of the qualitative analysis. In addition, there was an unexpected positive result for the co-researchers who were classmates. They strengthened their bond by sharing the research experience and reflecting on health and illness together. This two-phase approach made it possible to achieve our research goals and empower our co-researchers, while limiting the time invested for both adult and child researchers.

Use of videos in analysis

Our aim was active involvement of our co-researchers in interview analysis in an effective and efficient way. Data analysis in qualitative research is often a long and intensive process with large amounts of text. Locock et al. reported that reading the transcripts for young people in their study was difficult and inefficient. They concluded that it was more valuable to start a conversation and discuss the data rather than digging into the detailed transcripts.[10] Therefore, we decided to test other ways of involving young co-researchers in the analysis. Visuals such as photographs, drawings, or mapping methods are often used for collaboration with young children to collect data about children's views on, for example, the things they value in their lives.[25] Darbyshire et al. reported that using a variety of qualitative visual techniques was beneficial for getting children interested and engaged in research, and it provided a good way for children to express their views. There is a problem with using visuals as a participatory method rather than in analysis, as Darbyshire et al. pointed out: *"…having children take photographs and then having only adults 'interpret' (or possibly misinterpret) them is potentially an adultist approach to research on children that we sought to avoid"*.[26] Therefore, we used videos in the analysis stage to visualize the interview data that was to be analysed. Our study confirmed the benefits expressed by Darbyshire et al. The co-researchers liked the creative process when they developed the mind map, and the videos helped them to understand and empathize with the interviewees. Using the videos instead of transcripts made it more time efficient, while preserving the effectiveness of a thematic analysis. Reflecting on this, we realized that using videos rather than transcripts has another benefit regarding rigor of the qualitative data analyses. Analysis of interview data is often assumed to start at the moment that the interview has been fully transcribed, but even when this is verbatim, including descriptions of vocal emotions such as laughter, there will always be a loss of key elements such as volume of voices and facial expressions. This could present interviewees' experiences in a more abstract way than the original data show.[27, 28] In other words, by using videos, we might have started the analysis with a more authentic representation of our data.

For peer review only - http://bmjopen.bmj.com/site/about/guidelines.xhtml

Additional considerations and further research

Time investment is an important challenge for developing ways of involving children in research analysis. The method developed by Best et al. limited the analysis to two hours. Nevertheless, an additional time investment of four times 90-120 minutes was asked from participants for 'capacity building.' In these sessions, young people learned how to design and conduct a study, how to perform qualitative data analysis, and received an introduction into the subject of their data.[19] As described, we purposely did not train our co-researchers, both to avoid 'fitting' them into our own idea of what a qualitative researcher should be, and to limit time investment. Though we cannot make a comparison, the minimal training we provided to our co-researchers did not seem to have a negative impact on the result.

In addition to time investment, timing of research meetings should be considered. Many Young People's Advisory Groups (YPAGs) plan their meetings mostly during school holidays or on weekends.[29] INVOLVE identified parents and schools as a significant barriers to public involvement during school hours: *"…lack of schools' recognition of the value of their work sometimes acts as a barrier to them attending events which involve travel in school hours."*[14, p12] Nevertheless, we managed to set up a collaboration with a primary school for the second phase of our study, and planned the group meetings during school hours. The headmaster recognized the value of the research being carried out with children at school. For our co-researchers in the group meetings, participation during school time was a great way to be involved, since they reported they had busy schedules in their free time. One of our co-researchers said that being busy with homework for secondary school might be a reason not to participate in similar project in the future.

We recently started testing our two-phase approach with young people age 16 to 18, who will use this project for a school assignment, thereby creating a win-win situation. If this proves beneficial, we will consider starting a long-term collaboration with both primary schools and secondary schools to optimize collaboration between researchers and children, which could be beneficial for both individual researchers and the schools involved. We expect that a comparison of findings between adults, children, and young people will bring interesting new insights from different life experiences and ages of the researchers involved.

Study limitations

For peer review only - http://bmjopen.bmj.com/site/about/guidelines.xhtml

To test this new approach, we started with a small group of young co-researchers. A consequence is that we had to make a preselection of interviews from a larger dataset to explore in phase 1. Within this selection we aimed to have as much variation as possible, but we had to consider pragmatic matters as well, such as the length of the interviews. In addition, in our method children were not involved in making choices about specific quotes used in the results sections. As recruitment of co-researchers was a challenge, sampling was only based on age and on fluency in Dutch. However, a maximum variation sampling would be preferable to improve reflexivity.

CONCLUSION

Based on our study, we suggest that the two-phase approach provides an effective and efficient way to involve relatively young children in analysis of qualitative data by combining one-on-one meetings and group meetings. Presenting the interview data through videos rather than transcripts made it easier for children to understand the data, to empathize with the interviewees, and to limit time investment. The two-phase approach has the potential to prevent unrealistic interpretation of children's voices by adult researchers because it limits preselection of data by adults. Additional benefits are that children reflect on health and illness in their own lives, and they are empowered and engaged in medical research.

ETHICS APPROVAL

Both this study (M18.2334032 2018, July 24th) and our larger study (M16.192386 2016, May 10th) were concluded not to fall within the scope of the Medical Research Involving Human Subjects Act by the Medical Ethics Review Board of the University Medical Center Groningen.

ACKNOWLEDGEMENTS

We want to thank all participants in the original interview study, as well as our co-researcher participants from this project. Because this is a meta-analysis of the PPI project, we have decided not to invite the co-researchers as co-author on this article. Thanks to Laura Postma (PPIA2) for co-facilitating the group meetings.

FUNDING STATEMENT

This PPI study itself had low costs. Data collection for the main interview study was funded by the University of Groningen as part of the MD/PhD program of Malou Luchtenberg. This funding body has no role in the design of the study and collection, analysis, or interpretation of data, or in writing the manuscript.

COMPETING INTERESTS STATEMENT

None declared.

DATA SHARING STATEMENT

For peer review only - http://bmjopen.bmj.com/site/about/guidelines.xhtml

The original dataset with audio- and videotaped interviews is not available for the public due to privacy concerns.

AUTHOR'S CONTRIBUTION

ML, EM and EV designed the original study. ML was responsible for the data collection in phase one, and the group meetings were led by ML (PPIA1). ML made the first draft of the article, which was discussed regularly within the team and reviewed by EM and EV. All authors read and approved the final manuscript.

REFERENCES

1. World Medical Association. World Medical Association Declaration of Helsinki: ethical principles for medical research involving human subjects. *JAMA* 2013;310(20):2191–4. https://doi.org/10.1001/jama.2013.281053
2. Hennink M, Hutter I, Bailey A. Qualitative Research Methods. London: Sage 2011.
3. Taylor RM, Whelan JS, Gibson F, et al. Involving young people in BRIGHTLIGHT from study inception to secondary data analysis: insights from 10 years of user involvement. *Res Involv Engagem* 2018;4(1):1–14. https://doi.org/10.1186/s40900-018-0135-x
4. Jamal F, Langford R, Daniels P, et al. Consulting with young people to inform systematic reviews: an example from a review on the effects of schools on health. *Heal Expect* 2015;18(6):3225–35. https://doi.org/10.1111/hex.12312
5. Coad J, Evans R. Reflections on practical approaches to involving children and young people in the data analysis process. *Child Soc* 2008 Jan;22(1):41–52. https://doi.org/10.1111/j.1099-0860.2006.00062.x
6. Clavering EK, McLaughlin J. Children's participation in health research: from objects to agents? *Child Care Health Dev* 2010;36(5):603–11. https://doi.org/10.1111/j.1365-2214.2010.01094.x
7. Brady L-M, Preston J. Evaluating the extent and impact of young people's involvement in National Institute for Health Research (NIHR) studies: an assessment of feasibility. 2017 April. http://www.invo.org.uk/wp-content/uploads/2017/06/Impact-of-CYPs-involvement_JLI__GenR.pdf (accessed 01-07-2019)
8. Lundy L, McEvoy L, Byrne B. Working with young children as co-researchers: an approach informed by the United Nations Convention on the Rights of the Child. *Early Educ Dev* 2011;22(5):714–36. https://doi.org/10.1080/10409289.2011.596463
9. Holland S, Renold E, Ross NJ, et al. Power, agency and participatory agendas: a critical exploration of young people's engagement in participative qualitative research. *Childhood* 2010;17(3):360–75. https://doi.org/10.1177/0907568210369310
10. Locock L, Kirkpatrick S, Brading L, et al. Involving service users in the qualitative analysis of patient narratives to support healthcare quality improvement. *Res Involv Engagem* 2019;5(1):1–11. https://doi.org/10.1186/s40900-018-0133-z
11. INVOLVE. What is public involvement in research? *NHS Natl Inst Heal Res* 2015. http://www.invo.org.uk/find-out-more/what-is-public-involvement-in-research-2/ (accessed 10-07-2019)
12. Bird D, Culley L, Lakhanpaul M. Why collaborate with children in health research: an analysis of the risks and benefits of collaboration with children. *Arch Dis Child - Educ Pract Ed* 2013;98(2):42–8. https://doi.org/10.1136/archdischild-2012-303470
13. Preston J, Stones SR, Davies H, et al. How to involve children and young people in what is, after all, their research. *Arch Dis Child* 2019;104(5):494–500. https://doi.org/10.1136/archdischild-2018-315118

For peer review only - http://bmjopen.bmj.com/site/about/guidelines.xhtml

14. Sonpal K, Walker E, Swallow V, et al. Report on involving children and young people in research. Southampton: INVOLVE. 2019. https://www.invo.org.uk/wp-content/uploads/2019/06/77620-YPv3-Booklet-WEB.pdf (accessed 01-07-2019)
15. Blackburn S, McLachlan S, Jowett S, et al. The extent, quality and impact of patient and public involvement in primary care research: a mixed methods study. *Res Involv Engagem* 2018;4(1):1–18. https://doi.org/10.1186/s40900-018-0100-8
16. Domecq JP, Prutsky G, Elraiyah T, et al. Patient engagement in research: a systematic review. *BMC Heal Serv Res* 2014;14(89):1–9. https://doi.org/10.1186/1472-6963-14-89
17. Kellett M, Forrest R, Dent N, et al. "Just teach us the skills please, we'll do the rest": Empowering ten-year-olds as active researchers. *Child Soc* 2004;18(5):329–43. https://doi.org/10.1002/chi.807
18. Chappell P, Rule P, Dlamini M, et al. Troubling power dynamics: youth with disabilities as co-researchers in sexuality research in South Africa. *Childhood* 2014;21(3):385–99. https://doi.org/10.1177/0907568214525427
19. Best P, Badham J, Corepal R, et al. Network methods to support user involvement in qualitative data analyses: an introduction to Participatory Theme Elicitation. *Trials* 2017;18(1):1–14. https://doi.org/10.1186/s13063-017-2289-5
20. Dovey-Pearse G, Walker S, Fairgrieve S, et al. The burden of proof: The process of involving young people in research. *Helath Expectations* 2019;22:465-474. https://doi.org/10.1111/hex.12870
21. Brederoschool. Academische Basisschool 2019. Groningen, the Netherlands. https://brederoschool.openbaaronderwijsgroningen.nl/academischebasisschool/index.html (accessed 09-11-2018)
22. Dutch Medical Research Involving Human Subjects Act (WMO), 26 February 1998. https://wetten.overheid.nl/BWBR0009408/2019-04-02 (accessed 09-04-2019)
23. Cambridge University Press. Meaning of mind map in English. Cambridge Dictionary. 2019. https://dictionary.cambridge.org/dictionary/english/mind-map (accessed 19-09-2019)
24. Smith J, Firth J. Qualitative data analysis: the framework approach. Nurse Researcher 2011;18(2):52–62. https://doi.org/10.7748/nr2011.01.18.2.52.c8284
25. Coad J. Using art-based techniques in engaging children and young people in health care consultations and/or research. *J Res Nurs* 2007;12(5):487–97. https://doi.org/10.1177/1744987107081250
26. Darbyshire P, Macdougall C, Schiller W. Multiple methods in qualitative research with children: more insight or just more? *Qual Res* 2005;5(4):417–436. https://doi.org/10.1177/1468794105056921
27. Hewitt J. Ethical components of researcher-researched relationships in qualitative interviewing. *Qual Health Res* 2007;17(8):1149–59. https://doi.org/10.1177/1049732307308305
28. Alldred P, Gillies V. Eliciting research accounts: re/producing modern subjects. Ethics in Qualitative Research. London: Sage 2002:146–65.
29. Gaillard S, Malik S, Preston J, et al. Involving children and young people in clinical research through the forum of a European Young Persons' Advisory Group: needs and challenges. *Fundam Clin Pharmacol* 2018 Aug;32(4):357–62. https://doi.org/10.1111/fcp.12360

Figure 1. Overview of presented study embedded in larger context of research

For peer review only - http://bmjopen.bmj.com/site/about/guidelines.xhtml

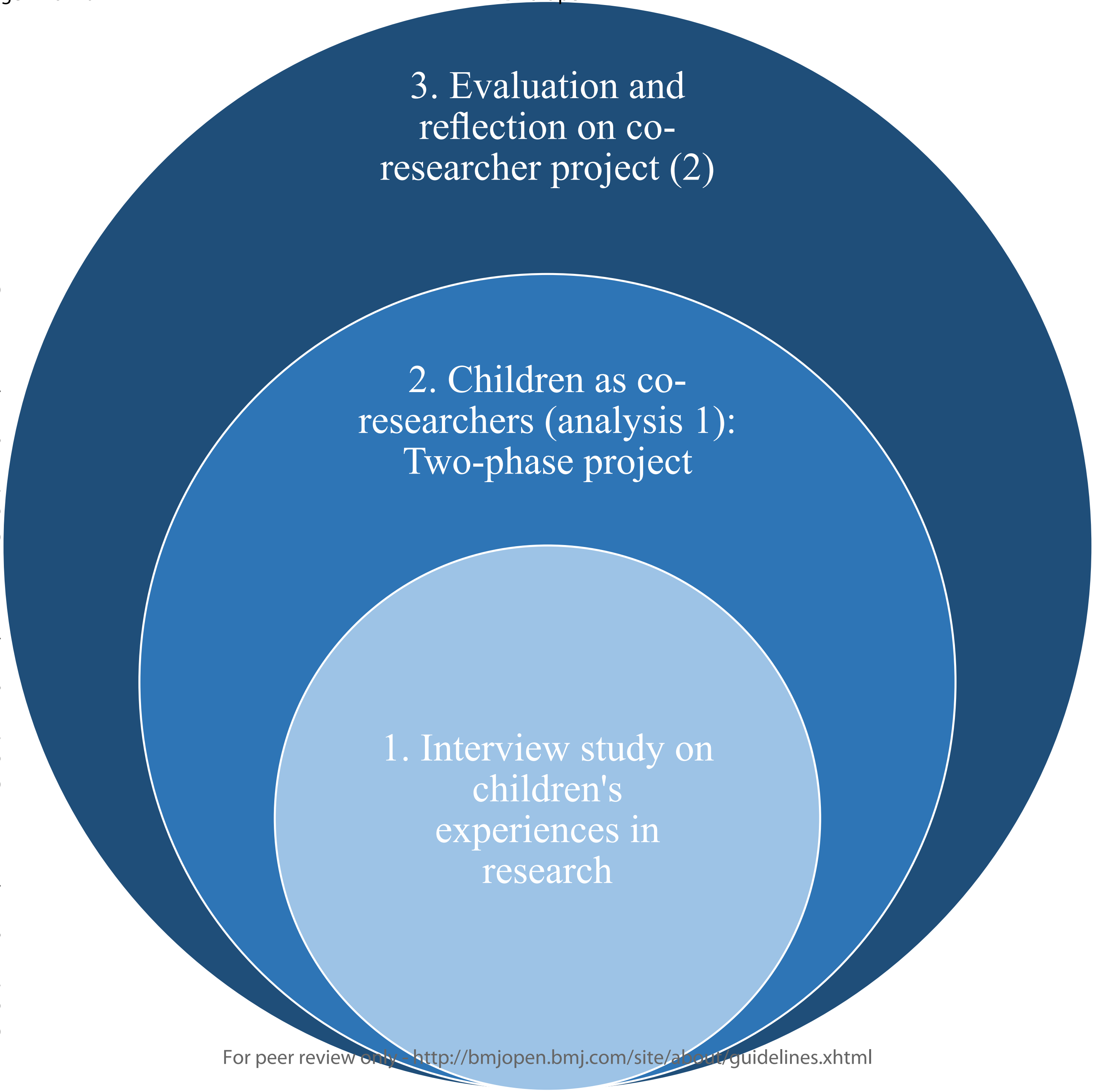
3. Evaluation and reflection on co-researcher project (2)
2. Children as co-researchers (analysis 1): Two-phase project
1. Interview study on children's experiences in research

‘I actually felt like I was a researcher myself.’ On Involving Children in the Analysis of Qualitative Paediatric Research

Journal:	*BMJ Open*
Manuscript ID	bmjopen-2019-034433.R2
Article Type:	Original research
Date Submitted by the Author:	17-Mar-2020
Complete List of Authors:	Luchtenberg, Malou; University of Groningen; University Medical Center Groningen, Department of Pediatrics<br>Maeckelberghe, Els; University of Groningen, Institute for Medical Education; University Medical Center Groningen<br>Verhagen, Eduard; University of Groningen; University Medical Center Groningen , Department of Pediatrics
**Primary Subject Heading**:	Paediatrics
Secondary Subject Heading:	Ethics, Qualitative research, Research methods
Keywords:	Involvement, Children, PAEDIATRICS, Patient Participation, Biomedical Research, QUALITATIVE RESEARCH

SCHOLARONE™ Manuscripts

I, the Submitting Author has the right to grant and does grant on behalf of all authors of the Work (as defined in the below author licence), an exclusive licence and/or a non-exclusive licence for contributions from authors who are: i) UK Crown employees; ii) where BMJ has agreed a CC-BY licence shall apply, and/or iii) in accordance with the terms applicable for US Federal Government officers or employees acting as part of their official duties; on a worldwide, perpetual, irrevocable, royalty-free basis to BMJ Publishing Group Ltd ("BMJ") its licensees and where the relevant Journal is co-owned by BMJ to the co-owners of the Journal, to publish the Work in this journal and any other BMJ products and to exploit all rights, as set out in our licence.

The Submitting Author accepts and understands that any supply made under these terms is made by BMJ to the Submitting Author unless you are acting as an employee on behalf of your employer or a postgraduate student of an affiliated institution which is paying any applicable article publishing charge ("APC") for Open Access articles. Where the Submitting Author wishes to make the Work available on an Open Access basis (and intends to pay the relevant APC), the terms of reuse of such Open Access shall be governed by a Creative Commons licence – details of these licences and which Creative Commons licence will apply to this Work are set out in our licence referred to above.

Other than as permitted in any relevant BMJ Author's Self Archiving Policies, I confirm this Work has not been accepted for publication elsewhere, is not being considered for publication elsewhere and does not duplicate material already published. I confirm all authors consent to publication of this Work and authorise the granting of this licence.

For peer review only

'I actually felt like I was a researcher myself.'

On Involving Children in the Analysis of Qualitative Paediatric Research

Malou Luchtenberg, Els Maeckelberghe, Eduard Verhagen

University of Groningen, University Medical Center Groningen

Malou L. Luchtenberg

Department of Pediatrics, Beatrix Children's Hospital

University of Groningen, University Medical Center Groningen, the Netherlands

Els L.M. Maeckelberghe

Institute for Medical Education

University of Groningen, University Medical Center Groningen, the Netherlands

A.A. Eduard Verhagen

Department of Pediatrics, Beatrix Children's Hospital

University of Groningen, University Medical Center Groningen, the Netherlands

Corresponding author:

Malou L. Luchtenberg

PO Box 30 001, 9700 RB Groningen

The Netherlands

m.l.luchtenberg@umcg.nl

+31 6 25 64 96 59

Word count: 4329 (excl. title page, abstract, references, tables and quotes)

Keywords: involvement, children, paediatrics, patient participation, biomedical research, qualitative research

For peer review only

For peer review only - http://bmjopen.bmj.com/site/about/guidelines.xhtml

ABSTRACT

Objectives
To evaluate the feasibility of a new approach to paediatric research whereby we involved children in analysing qualitative data, and to reflect on the involvement process.

Setting
This was a single-centre, qualitative study in the Netherlands. It consisted of research meetings with individual children at home (Phase 1) or group meetings at school (Phase 2). In Phase 1 we identified themes from a video interview during five one-on-one meetings between a child co-researcher and the adult researcher. In Phase 2, during two group meetings, we explored the themes in detail using fragments from 16 interviews.

Participants
We involved 14 school children (aged 10 to 14) as co-researchers to analyse children's interviews about their experience while participating in medical research. Notes were taken, and children provided feedback. A thematic analysis was performed using a framework approach.

Results
All co-researchers identified themes. The time needed to complete the task varied, as did the extent to which the meetings needed to be structured to improve concentration. The children rated time investment as adequate and they considered acting as co-researcher interesting and fun, adding that they had learnt new skills and gained new knowledge. The experience also led them to reflect on health matters in their own lives. The adult researchers considered the process relatively time intensive, but the project did result in a more critical assessment of their own work.

Conclusion
The new, two-phase approach of involving children to help analyse qualitative data is a feasible research method. The novelty lies in involving children to help identify themes from original interview data before exploring these themes in detail Preselection of data is thus limited. We recommend using videos rather than transcripts. Videos make it easier for children to understand the data and to empathise with the interviewees, and limits time investment.

STRENGTHS AND LIMITATIONS OF THIS STUDY

- This study describes a new approach to paediatric research whereby children are involved in analysing qualitative interview data. Its novelty lies in the fact that children are involved in helping to identify themes from original data as well as exploring the themes in more detail.
- This study explores the use of videos rather than transcripts to present the interviews to relatively young co-researchers.
- The study reflects on children's involvement as co-researchers from the perspective of the children themselves and from that of the adult researchers.
- In test phase presented here we limited the number of child co-researchers and selected the interviews from a larger dataset to include as much variation as possible.

For peer review only - http://bmjopen.bmj.com/site/about/guidelines.xhtml

INTRODUCTION

Researchers should be wary of interpretation bias when analysing data of qualitative studies. Qualitative research includes a subjective component that calls for reflexivity.[2] Because the life experiences and social situations of children differ from those of adults, their interpretations of data derived from interviews with children may differ from adults' interpretations. It is therefore desirable to involve children to strengthen the analysis of such qualitative data. There is little evidence in the literature on how children could be effectively involved in scientific data analysis.[3–10] Other challenges regarding patient and public involvement (PPI) [11] of children include lack of funding and lack of time, gatekeeping or power imbalances, and concerns about obtaining knowledge and training on how to involve children.[3,6,12–18] Measuring the impact of a PPI process on research output is difficult because the involvement process itself is complex and therefore its impact cannot by fully captured by evaluating outcomes.[19]

Best and colleagues recently introduced a new method called participatory theme elicitation, whereby a youth advisory panel is involved in qualitative data analysis. The method involves capacity building (training), data selection by adult researchers, data sorting by youth members, and final grouping and analysis by adult researchers.[20] A disadvantage of the method is that it involves data preselection by adults. We hypothesise that it is feasible to involve children from the beginning of data analysis, starting with the identification of themes from original data.

In a larger unpublished study, we collected the experiences of young people regarding their participation in medical research in order to provide recommendations for improving children's participation in research. In the present study, we aimed to explore whether it is feasible to involve children in the analysis of the qualitative data. We designed a two-phase approach that would be effective and efficient. Effective in the sense that it involved children to identify themes as well as to explore the themes in more detail and efficient in the sense of limiting time investment. We also aimed to reflect on the involvement process from the perspective of both adults and children. The results of the qualitative data analysis are to be published elsewhere.

Patient and public involvement

For peer review only - http://bmjopen.bmj.com/site/about/guidelines.xhtml

We explored the feasibility of an approach whereby children were involved in qualitative data analysis, both in helping adult researchers to identify themes from original data as in exploring the themes in more detail.

METHODS

This was a single-centre study performed by researchers of University Medical Center Groningen, the Netherlands. We investigated the involvement of children as co-researchers in identifying and analysing themes from interview data presented on video. We conducted the investigation in two phases: Phase 1 consisted of five individual meetings, and Phase 2 of two group meetings. In addition, we reflected on the children's involvement in the analysis process.

Recruitment and sampling

Phase 1

We approached potential co-researchers through national patient support organizations, primary schools, hospitals, social media, and by word-of-mouth. No research experience was required of the participants. Sampling was based on age (9-18 years) and participants were required to be fluent in Dutch because the meetings were held in that language. Initially, seven children volunteered. They each received an information leaflet. Even though recruitment was challenging we managed to achieve our goal of five participants in Phase 1.

Phase 2

We recruited the participants for Phase 2 in collaboration with a specific primary school in Groningen, the Netherlands, where teachers and learners are expected to display an academic mind-set aimed at research and analysis.[21] We invited one class of 15 children of the school's oldest leaners to participate. Ten learners volunteered, one of whom was unable to participate because of illness.

Informed consent

Phase 1

We supplied the potential participants with verbal and written information. We asked them to discuss the study with their parents and to reply by post, e-mail or by telephone. Before the session at the child's home started, he or she read the informed consent form and discussed it with the researcher and a parent. One parent was present throughout the session, but was

For peer review only - http://bmjopen.bmj.com/site/about/guidelines.xhtml

kindly asked to not interfere with the process. All the children, irrespective of age, were asked to sign the informed consent form to acknowledge that we appreciated their contribution equally. In accordance with the Dutch Medical Research Involving Human Subjects Act , parental consent was obtained in addition to the child's consent.[22] Children were also asked to sign a confidentiality agreement regarding any personal information present in the data they analysed. At the end of the session we gave the participants a €10 gift voucher as token of our appreciation of their time and a certificate acknowledging their contribution as co-researchers.

Phase 2

Two adult researchers visited the primary school to meet the learners, their teacher and headmaster, and to introduce the research project. The headmaster agreed to allow the children to participate during school hours. The potential participants received an information leaflet similar to the one given to the participants of Phase 1. We asked them to complete the consent form and the confidentiality agreement at home with a parent, and to return the form to their teacher. At the end of the group meetings we gave the participants a certificate. These children were not given a gift voucher because they participated during school hours.

Data characteristics

Data from the original interview study were analysed by our co-researchers. In addition, the adult researchers took notes during the analyses and written feedback was obtained from the participants before, during, and after participating. In Table 1 we provide additional information on the data collected for the original interview study. Out of a total of 23 interviews two were excluded because they were not videotaped. Another five were excluded because participants and parents had not consented to use the video data.

Table 1. Details of data of the original interview study on children's experiences in medical research

**Study characteristics**	
**Aim**	To explore children's experiences in medical research to obtain recommendations from their perspectives on how to improve children's involvement in research.
**Setting and research team**	Single-centre study conducted by a team of researchers at University Medical Center Groningen, the Netherlands. The research team consisted of an ethicist (EM), paediatrician (EV), and MD/PhD student (ML). All members were trained researchers and/or had previous experience in conducting qualitative research.

For peer review only - http://bmjopen.bmj.com/site/about/guidelines.xhtml

**Recruitment and sampling**	Recruitment through health providers from several hospitals, national patient support groups, social media, and by word-of-mouth. Purposive maximum variation sample: children, patients as well as healthy volunteers between 9 and 18 years old who were invited to participate in different types of medical research in the Netherlands and who either took part or declined to take part. The participants had no prior relationships with the members of the research team.
**Informed consent**	Informed consent given by one parent and the child or, in accordance to Dutch law, from 16 years and older by the child only.
**Data collection**	Twenty three semi-structured, in-depth interviews, lasting between 30 and 100 minutes, with children about their experiences in taking part in medical research, including recommendations for improvement of children's involvement in informed consent procedures and the research itself. A topic guide was developed based on a previous study in the United Kingdom. Interviews performed by ML took place at children's homes and were recorded on audio or video, transcribed verbatim, and returned to the participants. No comments from participants were received. Data collection continued until we reached data saturation of main themes.
**Ethical approval**	The conclusion of the Medical Ethics Review Board of the University Medical Center Groningen was that this study, no. M16.192386, 10 May 2016 fell beyond the scope of the Dutch Medical Research Involving Human Subjects Act .

The co-researchers received a brief interactive introduction to paediatric research and to the original interview study. They were asked to identify the main topics in video interviews and to summarise them on mind maps *(A type of diagram with lines and circles for organising information so that it is easier to use or remember*).[23, p1] To prevent the children from becoming 'little adult researchers' we did not train them extensively. Extensive training would also have been more time consuming for both the children and the researchers.

In Phase 1 the five co-researchers collaborated with the coordinating researcher (PPIA1 – PPI adult researcher 1 in a one-on-one session to identify the main themes in five different interviews. Together they watched a video of between 25 to 45 minutes of another young person and discussed the emerging themes. This took place at the participants' home and lasted between two and three and a half hours. During Phase 1 the adult researcher spent a total of eleven hours travelling to and from co-researchers homes.

During the two group meetings in Phase 2, we explored in detail two themes that had been identified during Phase 1. For this purpose we compiled two five-minute videos from fragments of several interviews from the dataset of the original study. The group meetings took place at a local primary school and lasted approximately two and a half hours. Travelling time for adult researchers was two hours, including the introductory meeting when we handed out the information sheets. In addition to travelling time, the time investment of adult

For peer review only - http://bmjopen.bmj.com/site/about/guidelines.xhtml

researchers was approximately five hours. This included the time spent preparing the instructions and making the compilation videos. The material costs of the project were low and the travelling costs minimal.

The aim of the analysis process in both phases was to identify the main themes in the video through open, unstructured discussions with the child co-researchers. To facilitate interaction and discussion the co-researchers and the adult researchers could pause the video at any time. They took notes of what they thought the interviewee found important. PPIA1 and PPIA2 allowed moments of personal reflection, but were on guard for potential intertwining of co-researchers' personal experiences with interviewees' experiences. After watching the video the co-researchers drew a mind map depicting their interpretation of the connection between different themes.[23] The mind maps were drawn on A3-sized sheets of paper, using different sizes of sticky notes, and coloured pens. Besides, the participants were free to use materials of their own choice. The researcher asked the participants questions about the importance of certain themes, the identification of overlapping themes, and the reason why they had chosen a certain theme. The participants led the discussion and made the final decision in case of a disagreement about a theme.

Data collection and analysis

Before the study actually commenced, we asked the child co-researchers why they would like to take part. The adult researchers took notes of how participants fulfilled their role as co-researcher, and how the participants reflected on this process. Child co-researchers completed a feedback form after the analysis (Table 2). In addition, we briefly evaluated the process orally. All meetings were recorded on audio tape.

We performed a thematic analysis using a framework approach.[24] Familiarisation and initial theme identification was done by PPIA1 (ML) and discussed with EM (individual meetings) and PPIA2 (group meetings) based on the audio tape, notes, and the participants' written feedback. Some themes, such as time investment, were identified in advance from the literature, others were derived from the data. Themes were refined and conceptualised during regular meetings with the research team and any disagreements were discussed and a final decision reached by consensus. Because the sample for this exploratory study was relatively small , we did not aim for data saturation.

For peer review only - http://bmjopen.bmj.com/site/about/guidelines.xhtml

Table 2. Feedback form

1. Did you understand beforehand what your role was in the project? (No / a little / yes)
2. How could we improve the information about working as a co-researcher?
3. What was it like for you to work as a co-researcher?
   a. positive aspects
   b. points of improvement
4. Did you learn anything from being a co-researcher? If so, what did you learn?
5. Would you like to be a co-researcher more often?
6. Would you recommend other children to become co-researchers? Why?
7. How would you rate your time investment? (Too long, adequate, too short)
8. a. What did you think of the EUR 10 gift voucher? (Phase 1)
   b. What did you think about participating in this project during school hours? Why? (Phase 2)
9. Do you have suggestions for improving this evaluation form?

RESULTS

Participant characteristics

Fourteen children, eight girls and six boys, participated as co-researchers in this study. Two participants had experience because they had been chronic patients themselves. None of the children had been a co-researcher before. Tables 3 and 4 show the characteristics of the child and adult participants.

Table 3. Child participant characteristics

**Characteristics of child co-researchers**	**N* (%)****
Sex	
Girl	8 (57)
Boy	6 (43)
Age	
10 years	1 (7)
11 years	10 (71)
12 years	2 (14)
13 years	0 (0)
14 years	1 (7)
School attended	
Primary School	13 (93)
Secondary School	1 (7)
Hospital/Disease experience (lived experience)	
Currently suffering from a disease	2 (14)
Hospitalization or minor surgery in the past	6 (43)
Family member(s) who suffer from a disease	2 (14)
None	4 (29)
Research experience as participant	
Yes	2 (14)
No	12 (86)
Experience as co-researcher	
Yes	0 (0)
No	14 (100)

* Number of child participants; N = 14
** Rounded to nearest whole number

For peer review only

For peer review only - http://bmjopen.bmj.com/site/about/guidelines.xhtml

Table 4. Adult participants characteristics

Participant	Age	Sex	Experience illness /hospital	Research experience (participating in research)	Research experience (performing research)
PPIA1	27	Woman	As a medical student. Graduated as medical doctor in August 2019.	Yes. Participant in two large cohort studies for several years.	Training and experience in qualitative research for PhD.
PPIA2	23	Woman	As a medical student. Started internships in September 2019.	No previous experience in medical research participation.	Trained in qualitative research as a former psychology student.

Reflection and evaluation of the involvement process

The results can be divided into five main themes: (1) understanding the study procedures, (2) empowerment, (3) reflection on health and illness, (4) interest in the bigger picture, and (5) reflection on time investment. The results of the feedback form are summarised in Table 5.

Table 5. Summary of written feedback

Theme (question from Table 2)	Summary of written feedback	N* (%)**
**Understanding the study procedures** (1,2,9)	*Understanding their role as co-researcher before start****	
	No	0 (0)
	A little	12 (86)
	Yes	2 (14)
	Suggestions for improving the information about working as co-researcher	
	Don't know	
	Everything was clear	4 (29)
	Use fewer difficult words	5 (36)
	Explain that we had to take notes and create a mind map	4 (29)
		1 (7)
	Suggestions for improving the feedback form of the co-researcher project	
	Adding a question about the overall experience	1 (7)
	No recommendations	13 (93)
**Empowerment** (3-6)	*Positive experience as co-researcher*	
	Fun	14 (100)
	Interesting	4 (29)
	Helping other children	1 (7)
	Learning something new	1 (7)
	Time investment was okay	2 (14)
	Receiving a certificate	1 (7)
	Points of improvement for co-researcher project	
	No points of improvement	12 (86)
	Shorter interviews	1 (7)
	The project should take the whole school day (instead of a half one)	1 (7)
	Lessons learnt from being a co-researcher	
	Taking notes	2 (14)
	Critical thinking and listening	4 (29)
	About a medical condition	2 (14)
	About doing research	2 (14)
	About how children think and feel about research	2 (14)
	That children think differently from adults	1 (7)

For Peer review only

For peer review only - http://bmjopen.bmj.com/site/about/guidelines.xhtml

	That it is fun and that you learn a lot<br>Not really	1 (7)<br>1 (7)
	Would like to be co-researcher more often including reason<br>Yes, because it's fun<br>Yes, because it's interesting<br>Yes, because I like to help people<br>Yes, I know what to expect now<br>Yes, if it doesn't hurt<br>It is fun, but depends on how much time I have	 12 (86)<br>5 (36)<br>1 (7)<br>1 (7)<br>1 (7)<br>1 (7)
	Would you recommend others to become co-researcher?<br>Yes, because it's (super) fun<br>Yes, because it's interesting/you learn something from it<br>Yes, because you receive a gift voucher<br>Yes, because you can help other people<br>Yes, because you get sweets<br>Yes, but it depends on whether it suits them	 9 (64)<br>7 (50)<br>1 (7)<br>2 (14)<br>1 (7)<br>1 (7)
**Time investment** (7,8b)	*Rating of time investment****<br>Too long<br>Adequate<br>Too short	 1 (7)<br>12 (86)<br>1 (7)
	*Thoughts on having this project during school time (Phase 2, n=9)*****<br>Fun/good, because you didn't have to do schoolwork<br>Fun/good, because you don't miss free time after school<br>Don't mind	 6 (67)<br>4 (44)<br>1 (11)
**Compensation** (8b)	*Thoughts on receiving a gift voucher (Phase 1, n=5)*****<br>Fun/good<br>Not necessary<br>Creative	 5 (100)<br>2 (40)<br>1 (20)

* Number of child participants; N = 14. Some participants provided more than one answer
** Rounded to nearest whole number
*** The feedback provided is based on a multiple choice question
**** Calculation based on a sub-selection of total participants, because children took part in a different phase

1. Understanding of study procedures

When we asked the participants in the group meetings what they expected of the session, they remembered that the main idea was '*doing research about research.*' Nonetheless, not everyone remembered the details such as whether photographs would be taken. Even though the information was especially written with children in mind, the participants recalled that it was mostly read and signed by their parents, and had not always been discussed with them. In the case of the co-researchers in the individual meetings this was different. Here the researcher and the parents were present and encouraged the children to complete the form themselves. Parents were available in the background in case their help was needed.
Most children reported that they had a general idea about what their role was in the project. We explained their role to them in detail during the actual project. One of them explained:

> *'I already understood it but once you are doing it you understand it [better].'* (Girl, 11 years old, individual meeting)

For peer review only - http://bmjopen.bmj.com/site/about/guidelines.xhtml

2. Empowerment

By involving children as co-researchers they gained knowledge, they learnt new skills, and they became more confident in fulfilling their role during the analysis process. This was mostly because they enjoyed the new experience of contributing to research or helping others. Children felt empowered by working as a co-researcher. One of them explained it as:

> *'It actually felt a bit like I was a researcher myself.'* (Girl, 11 years old, group meeting)

They reported gaining new knowledge about certain health matters and they realised what it is like to be ill. They learnt how to do research, how to think critically, and how to take notes. Here are some representative answers given:

> *'You have to think carefully before you draw conclusions.'* (Boy, 11 years old, group meeting)

> *'A bit about how ill children felt afterwards [after participating in research].'* (Girl, 11 years old, group meeting)

All the participants were positive about the idea of being a co-researcher more often, mostly for similar reasons as for wanting to take part in the first place:

> *'Yes, it was fun, relaxed, and instructive.'* (Girl, 11 years old, group meeting)

One participant, the only one attending secondary school, mentioned it would depend on whether he had the time to take part because of homework and sports activities in his free time. All participants reported that they would recommend others to become co-researchers, though one participant acknowledged that it might not suit everyone. She mentioned that some children might not enjoy it or might not have the skills to do such work.

During the analysis process in Phase 1, the co-researchers grew noticeably more confident as time progressed. The adult researcher retreated to the background and stimulated the co-researchers to take the lead, which most of them did eventually. One participants actually

For peer review only - http://bmjopen.bmj.com/site/about/guidelines.xhtml

asked the researcher questions about her observations, instead of the other way around. Most participants, however, needed some form of structuring support from the adult researcher throughout.

During the group meetings the support needed from the adult researcher was different. The co-researchers needed more structuring because of group dynamics. Chaos ensued when several co-researchers started talking at the same time, and shyer children tended to not be heard. Interestingly, the two groups chose different ways of translating their notes to the mind map. In one group the adult researcher noticed a clear distinction in participants' role preferences, and the co-researchers divided the roles between themselves. Some preferred an executive role, such as writing down themes on the 'sticky notes', while others preferred to simply express their ideas and to play a more coordinating role. Some found it difficult to summarise their notes and suggested first underlining important notes:

> *'We could also just first underline what we think is important.'* (Girl, 11 years old, group meeting)

The co-researchers in the other group together decided that they all wanted to write down their own notes on 'sticky notes' and to put them all on the mind map. The outcome was a mind map that displayed different topics as well as providing insight into how important the individuals thought a certain topic was by the number of 'sticky notes' on the same topic. Others only needed a bit more time and space to find their own role.

It was challenging for the adult researchers to not provide answers themselves when the participants indicated that they did not know how to proceed with the analysis. By repeating or rephrasing their question and by acknowledging that they were doing the right thing, the adult researcher could reinforce the children. Both adult researchers were surprised by the co-researcher's achievements. Throughout the project the co-researchers displayed the ability to identify themes and to visualise them in mind maps, underlining the feasibility of this approach and its value for interpreting data.

The researchers learnt a great deal from involving children in the analyses because the participants were very open and direct in their feedback. If they did not understand something,

For peer review only - http://bmjopen.bmj.com/site/about/guidelines.xhtml

for example, if a question was not clearly formulated, or if they did not understand medical jargon such as 'treatment protocol', they said so immediately.

Whereas the adult researchers tended to generalise findings, the co-researchers stuck more to the original data. One topic, for example, dealt with recommendations to researchers for improving young people's experiences when participation in medical research. The interviewees mentioned issues like making hospital visits more enjoyable and gave concrete suggestions. All the researchers, adults and children alike, started from the original data, but there appeared to be a difference in analysis. The concrete suggestions in the children's analyses remained and made their recommendations were therefore emphasised, while the adults generalised them into 'ways to brighten up the visits'. The co-researchers brought the adults back to the basics that were important to children.

3. Reflection on health and illness

The participants empathised with the young people who shared their experiences in the video. They wondered whether they were still ill or asked if they still lived and hoped they were all right. The following is a representative example:

> *'None of these children [in the video] is deadly ill, right?'* (Girl, 11 years old, group meeting)

The adult researcher explained the different illnesses the interviewees had, and mentioned that some of them had been critically ill, for example, with leukaemia, but that they were stable at the time they were interviewed.

The co-researchers asked questions about the illnesses and what the consequences might be for the lives of the children such as two siblings with a hereditary condition. They also shared their own experiences, for example, about relatives who had cancer.

4. Interest in the bigger picture

Even though we only involved the participants in the analysis stage of the main interview study, they were well aware that this was part of a bigger study. They asked about the other participants and previous experiences with involving children in research analysis, and wondered whether we would show the mind maps they had made to the interviewees.

For peer review only - http://bmjopen.bmj.com/site/about/guidelines.xhtml

Some of the questions they asked were:

> *'Have you been to children's homes?' 'Are you also doing this [project] at other schools?' 'How many times have you done this?'* (Several co-researchers in the focus groups, boys and/or girls, 11 or12 years old, not clearly identifiable from the audio)

Hearing that they were the first group of children that participated as co-researchers in this project made them feel special. They expressed the wish to receive the final results and hoped we would do this project again. The co-researchers had a broader interest than just fulfilling their role as co-researchers. They also asked personal questions, such as why the adult researchers did this research project and whether it was part of their university training. They also acknowledged and enjoyed helping the adult researchers with their research:

> *'It is, off course, good for you [adult researchers] that we participate so that you can continue doing research about research of the research.'* (Girl, 11 years old, group meeting)

5. Reflection on time investment

Most children said the time investment was appropriate. One child in the group meetings reported that a shorter meeting would have been better because some children became distracted:

> '*Because at the end we were chatting a bit, [we got] distracted.' [Boy, 11 years old, group meeting]*

This was also observed by the adult researchers. Another participant, however, reported that he would have like the session to last longer because he really liked it and did not want to return to his normal schoolwork. The Phase 2 participants all reported that it was an interesting and fun alternative to normal school tasks. They thought it was good to do the project during school time because this way they would not miss out on any free time. One of the participants explained this:

> *'It was fun because you didn't have to work, and if it hadn't been during school it was inconvenient.' (Girl, 11 years old)*

For peer review only - http://bmjopen.bmj.com/site/about/guidelines.xhtml

From the adult perspective, time was invested in recruitment, developing material to introduce and explain the procedure, and thinking about how to best involve children. The actual analysis with co-researchers was time-intensive and lasted longer than we had expected. Nevertheless, the time invested was considered reasonable given the empowerment of children, their learning new skills, and their views on our data provided additional insight, which is promising for the interpretation of our interviews.

DISCUSSION

Little evidence is available on how to involve children in research.[12] In this paper we describe how we involved relatively young children in the analysis of medical research interviews analysis using a two-phase approach. We deployed various strategies to avoid a tokenistic approach, to address challenges regarding time management, and to empower children during the process.

Two-phase approach

Involving relatively young children, aged 10 to14, in our interview analyses was a challenging process. Our aim was to limit preselection of data by adults by introducing one-on-one meetings during which research interviews were analysed by young co-researchers. This approach could be considered an extension of Best's 'participatory theme elicitation' method.[20] Even though the sessions lasted longer than originally intended, the fact that we could focus on one individual worked to our advantage. In our opinion the project benefitted from the fact that the individual meetings were held at the children's homes, which constituted a safe and familiar environment. This confirms findings from Dovey-Pearce and colleagues who highlighted the importance of face-to-face meetings to establish relationships.[19] The themes identified during Phase 1 were explored in detail during group meetings in Phase 2. Working with a number of co-researchers in this phase improved the rigor of the qualitative analysis. In addition, there was an unexpected positive result for the co-researchers who were classmates. Their bond was strengthened by their shared research experience and by reflecting on health and illness together. The two-phase approach enabled us to achieve our research goals and to empower our co-researchers, while keeping within reasonable the time limits. This applied to adult and child researchers alike.

Use of videos in analysis

For peer review only - http://bmjopen.bmj.com/site/about/guidelines.xhtml

Our aim was to involve child co-researchers in interview analyses in an effective and efficient way. Data analysis in qualitative research is often lengthy process involving large quantities of text. Locock and colleagues reported that young people reading through the transcripts was tedious and inefficient. They concluded that it was more effective to discuss the data rather than digging into detailed transcripts.[10] We decided to explore other ways of involving young co-researchers in interview analyses. Visuals such as photographs, drawings, or mapping methods are often used in collaborations with young children to collect data about children's views on, for example, what they value in their lives.[25] Darbyshire and colleagues reported that using a variety of qualitative visual techniques is helpful for engaging children in research. It also provides a good way for children to express their views. There is, however, a problem with using visuals as a participatory method rather than in analysis, as Darbyshire and colleagues pointed out: *'…having children take photographs and then having only adults "interpret" (or possibly misinterpret) them is potentially an adultist approach to research on children that we sought to avoid.'*[26] For this reason we used videos in the analysis stage to visualise the interview data to be analysed. Our study confirmed the benefits expressed by Darbyshire and colleagues. The co-researchers enjoyed the creative process of developing the mind map and the videos helped them understand and empathise with the interviewees. Using videos rather than transcripts made the process more time-efficient, while preserving the effectiveness of a thematic analysis. Another benefit of videos over transcripts concerned the rigor of the qualitative data analyses. The analysis of interview data is often assumed to start as soon as the interview has been fully transcribed but, even in case of a verbatim transcript including descriptions of vocal emotions such as laughter, the loss of key elements, such as volume of voices and facial expressions, remains. This could present interviewees' experiences in a more abstract way than the original data show.[27, 28] Put differently, by using videos we possibly started the analysis with a more authentic representation of the data.

Additional considerations and further research

Time investment is an important consideration when developing ways of involving children in research analysis. In the method developed by Best and colleagues the analysis is limited to two hours. An additional time investment of four times 90 to 120 minutes is asked of participants for 'capacity building.' In these sessions, young people learn how to design and conduct a study, how to perform qualitative data analyses, and they receive an introduction into the subject matter of their data.[20] We purposely did not train our co-researchers to

For peer review only - http://bmjopen.bmj.com/site/about/guidelines.xhtml

avoid shaping them to comply with our idea of a qualitative researcher, and to limit time investment. Though we cannot make a comparison, the little training we gave our co-researchers did not seem to have had a negative impact on the result.

In addition to time investment, timing of research meetings should be considered. Many Young People's Advisory Groups generally plan their meetings during school holidays or weekends.[29] The INVOLVE Advisory Group of the United Kingdom's National Institute for Health Research, which supports active public involvement in the National Health Service, public health, and social care research,[30] identified parents and schools as a significant barrier to public involvement during school hours: *...lack of schools' recognition of the value of their work sometimes acts as a barrier to them attending events which involve travel in school hours.* [14, p12] Nevertheless, thanks to a cooperative headmaster, we managed to set up a collaboration with a primary school for Phase 2 of our study, and planned the group meetings during school hours. For our co-researchers in the group meetings, participating during school time was preferable to after school, because they reported that they had busy schedules or had to do homework during their free time.

Our results showed that children tend to include more concrete topics in their analyses, whereas adults analyse data in a more abstract way. This is in line with the cognitive development of children, who transform from concrete to abstract conceptualisation later in adolescence.[31, 32] Consequently, we expect that an evaluation of the data analysis process performed by children, young people, and adults will provide additional interesting insights. Recently, we started testing our two-phase approach with young people aged 16 to 18. They will use this project for a school assignment, thereby creating a situation that is mutually beneficial. If this proves successful, we consider setting up a long-term collaboration with primary schools and secondary schools to optimise collaboration between researchers and children to help decrease the knowledge gap between academia and society.

Study limitations

To test this new approach we started with a small group of young co-researchers. As a consequence, we had to select of interviews from a larger dataset to analyse in Phase 1. We aimed for as much variation as possible within this selection, but we also needed to be pragmatic, regarding the length of the interviews for instance. In addition, in our method children were not involved in making choices about specific quotes used in the results

For peer review only - http://bmjopen.bmj.com/site/about/guidelines.xhtml

sections. As recruiting co-researchers was challenging, sampling was limited to age and to fluency in the Dutch language. Maximum variation sampling would, however, be preferable to improve reflexivity.

CONCLUSION

We suggest that the two-phase approach to involving young children in analysing qualitative data is feasible. Its novelty lies in recruiting children to help identify themes from original data before the themes are explored in detail. Thus preselection of data by adults is limited. By combining one-on-one meetings and group meetings the two-phase approach is an effective and efficient way of involving relatively young children in analysing qualitative data. Additional benefits are that children reflect on health and illness in their own lives, they are empowered, and engaged in medical research. We recommend presenting the interview data on videos rather than through transcripts. Videos make it easier for children to understand the data, to empathise with the interviewees, and it limits time investment.

ETHICS APPROVAL

The Medical Ethics Review Board of the University Medical Center Groningen concluded that neither this study (M18.2334032, 24 July 2018) nor our larger study (M16.192386, 10 May 2016) fall within the scope of the Dutch Medical Research Involving Human Subjects Act.

ACKNOWLEDGEMENTS

We thank all the participants in the original interview study and our co-researcher participants in this project. Because this is a meta-analysis of the PPI project, we decided not to invite the co-researchers as co-author of this article. We thank Laura Postma (PPIA2) for helping to facilitate the group meetings.

FUNDING STATEMENT

The PPI study itself was a low-cost project. Data collection for the main interview study was funded by the University of Groningen as part of the MD/PhD program of Malou Luchtenberg. This funding body has no role in the design of the study and collection, analysis, or interpretation of data, or in writing the manuscript.

COMPETING INTERESTS STATEMENT

None declared.

DATA SHARING STATEMENT

The original dataset with audiotaped and videotaped interviews is not available for the public on account of privacy concerns.

AUTHOR'S CONTRIBUTION

ML, EM and EV designed the original study. ML was responsible for the data collection in Phase 1 and the group meetings were led by ML (PPIA1). Analysis was performed by ML,

For peer review only - http://bmjopen.bmj.com/site/about/guidelines.xhtml

checked by EM, and discussed with EV. ML made the first draft of the article, which was subsequently discussed within the team and reviewed by EM and EV. All authors read and approved the final manuscript.

REFERENCES

1. World Medical Association. World Medical Association Declaration of Helsinki: ethical principles for medical research involving human subjects. *JAMA* 2013;310(20):2191–4. https://doi.org/10.1001/jama.2013.281053
2. Hennink M, Hutter I, Bailey A. The nature of qualitative research. Subjectivity and the need for reflexivity. In: Qualitative Research Methods. London: Sage 2011:19-23.
3. Taylor RM, Whelan JS, Gibson F, et al. Involving young people in BRIGHTLIGHT from study inception to secondary data analysis: insights from 10 years of user involvement. *Res Involv Engagem* 2018;4(1):1–14. https://doi.org/10.1186/s40900-018-0135-x
4. Jamal F, Langford R, Daniels P, et al. Consulting with young people to inform systematic reviews: an example from a review on the effects of schools on health. *Heal Expect* 2015;18(6):3225–35. https://doi.org/10.1111/hex.12312
5. Coad J, Evans R. Reflections on practical approaches to involving children and young people in the data analysis process. *Child Soc* 2008 Jan;22(1):41–52. https://doi.org/10.1111/j.1099-0860.2006.00062.x
6. Clavering EK, McLaughlin J. Children's participation in health research: from objects to agents? *Child Care Health Dev* 2010;36(5):603–11. https://doi.org/10.1111/j.1365-2214.2010.01094.x
7. Brady L-M, Preston J. Evaluating the extent and impact of young people's involvement in National Institute for Health Research (NIHR) studies: an assessment of feasibility. 2017 April. http://www.invo.org.uk/wp-content/uploads/2017/06/Impact-of-CYPs-involvement_JLI__GenR.pdf (accessed 01-07-2019)
8. Lundy L, McEvoy L, Byrne B. Working with young children as co-researchers: an approach informed by the United Nations Convention on the Rights of the Child. *Early Educ Dev* 2011;22(5):714–36. https://doi.org/10.1080/10409289.2011.596463
9. Holland S, Renold E, Ross NJ, et al. Power, agency and participatory agendas: a critical exploration of young people's engagement in participative qualitative research. *Childhood* 2010;17(3):360–75. https://doi.org/10.1177/0907568210369310
10. Locock L, Kirkpatrick S, Brading L, et al. Involving service users in the qualitative analysis of patient narratives to support healthcare quality improvement. *Res Involv Engagem* 2019;5(1):1–11. https://doi.org/10.1186/s40900-018-0133-z
11. INVOLVE. What is public involvement in research? *NHS Natl Inst Heal Res* 2015. http://www.invo.org.uk/find-out-more/what-is-public-involvement-in-research-2/ (accessed 10-07-2019)
12. Bird D, Culley L, Lakhanpaul M. Why collaborate with children in health research: an analysis of the risks and benefits of collaboration with children. *Arch Dis Child - Educ Pract Ed* 2013;98(2):42–8. https://doi.org/10.1136/archdischild-2012-303470
13. Preston J, Stones SR, Davies H, et al. How to involve children and young people in what is, after all, their research. *Arch Dis Child* 2019;104(5):494–500. https://doi.org/10.1136/archdischild-2018-315118
14. Sonpal K, Walker E, Swallow V, et al. Report on involving children and young people in research. Southampton: INVOLVE. 2019. https://www.invo.org.uk/wp-content/uploads/2019/06/77620-YPv3-Booklet-WEB.pdf (accessed 01-07-2019)
15. Blackburn S, McLachlan S, Jowett S, et al. The extent, quality and impact of patient

For peer review only - http://bmjopen.bmj.com/site/about/guidelines.xhtml

and public involvement in primary care research: a mixed methods study. *Res Involv Engagem* 2018;4(1):1–18. https://doi.org/10.1186/s40900-018-0100-8
16. Domecq JP, Prutsky G, Elraiyah T, et al. Patient engagement in research: a systematic review. *BMC Heal Serv Res* 2014;14(89):1–9. https://doi.org/10.1186/1472-6963-14-89
17. Kellett M, Forrest R, Dent N, et al. "Just teach us the skills please, we'll do the rest": Empowering ten-year-olds as active researchers. *Child Soc* 2004;18(5):329–43. https://doi.org/10.1002/chi.807
18. Chappell P, Rule P, Dlamini M, et al. Troubling power dynamics: youth with disabilities as co-researchers in sexuality research in South Africa. *Childhood* 2014;21(3):385–99. https://doi.org/10.1177/0907568214525427
19. Dovey-Pearse G, Walker S, Fairgrieve S, et al. The burden of proof: The process of involving young people in research. *Helath Expectations* 2019;22:465-474. https://doi.org/10.1111/hex.12870
20. Best P, Badham J, Corepal R, et al. Network methods to support user involvement in qualitative data analyses: an introduction to Participatory Theme Elicitation. *Trials* 2017;18(1):1–14. https://doi.org/10.1186/s13063-017-2289-5
21. Brederoschool. Academische Basisschool 2019. Groningen, the Netherlands. https://brederoschool.openbaaronderwijsgroningen.nl/academischebasisschool/index.html (accessed 09-11-2018)
22. Dutch Medical Research Involving Human Subjects Act (WMO), 26 February 1998. https://wetten.overheid.nl/BWBR0009408/2019-04-02 (accessed 09-04-2019)
23. Cambridge University Press. Meaning of mind map in English. Cambridge Dictionary. 2019. https://dictionary.cambridge.org/dictionary/english/mind-map (accessed 19-09-2019)
24. Smith J, Firth J. Qualitative data analysis: the framework approach. Nurse Researcher 2011;18(2):52–62. https://doi.org/10.7748/nr2011.01.18.2.52.c8284
25. Coad J. Using art-based techniques in engaging children and young people in health care consultations and/or research. *J Res Nurs* 2007;12(5):487–97. https://doi.org/10.1177/1744987107081250
26. Darbyshire P, Macdougall C, Schiller W. Multiple methods in qualitative research with children: more insight or just more? *Qual Res* 2005;5(4):417–436. https://doi.org/10.1177/1468794105056921
27. Hewitt J. Ethical components of researcher-researched relationships in qualitative interviewing. *Qual Health Res* 2007;17(8):1149–59. https://doi.org/10.1177/1049732307308305
28. Alldred P, Gillies V. Eliciting research accounts: re/producing modern subjects. Ethics in Qualitative Research. London: Sage 2002:146–65.
29. Gaillard S, Malik S, Preston J, et al. Involving children and young people in clinical research through the forum of a European Young Persons' Advisory Group: needs and challenges. *Fundam Clin Pharmacol* 2018 Aug;32(4):357–62. https://doi.org/10.1111/fcp.12360
30. National Institute for Health Research. About INVOLVE. https://www.invo.org.uk/about-involve/ (accessed 04-03-2020)
31. Garrison W, Felice ME. Adolescence. In: Carey WB, Crocker AC, Coleman WL, Elias ER, Feldman HM, eds. Developmental-Behavioral Pediatrics (Fourth Edition) 2009:62–73. https://doi.org/https://doi.org/10.1016/B978-1-4160-3370-7.00006-7
32. Christie D, Viner R. Adolescent development. *BMJ (Clinical Research Ed.)* 2005;330:301. https://doi.org/10.1136/bmj.330.7486.301

For peer review only - http://bmjopen.bmj.com/site/about/guidelines.xhtml

For peer review only

BMJ Open

'I actually felt like I was a researcher myself.' On Involving Children in the Analysis of Qualitative Paediatric Research in the Netherlands

Journal:	*BMJ Open*
Manuscript ID	bmjopen-2019-034433.R3
Article Type:	Original research
Date Submitted by the Author:	04-Jun-2020
Complete List of Authors:	Luchtenberg, Malou; University of Groningen; University Medical Center Groningen, Department of Pediatrics<br>Maeckelberghe, Els; University of Groningen, Institute for Medical Education; University Medical Center Groningen<br>Verhagen, Eduard; University of Groningen; University Medical Center Groningen , Department of Pediatrics
**Primary Subject Heading**:	Paediatrics
Secondary Subject Heading:	Ethics, Qualitative research, Research methods
Keywords:	Involvement, Children, PAEDIATRICS, Patient Participation, Biomedical Research, QUALITATIVE RESEARCH

SCHOLARONE™
Manuscripts

I, the Submitting Author has the right to grant and does grant on behalf of all authors of the Work (as defined in the below author licence), an exclusive licence and/or a non-exclusive licence for contributions from authors who are: i) UK Crown employees; ii) where BMJ has agreed a CC-BY licence shall apply, and/or iii) in accordance with the terms applicable for US Federal Government officers or employees acting as part of their official duties; on a worldwide, perpetual, irrevocable, royalty-free basis to BMJ Publishing Group Ltd ("BMJ") its licensees and where the relevant Journal is co-owned by BMJ to the co-owners of the Journal, to publish the Work in this journal and any other BMJ products and to exploit all rights, as set out in our licence.

The Submitting Author accepts and understands that any supply made under these terms is made by BMJ to the Submitting Author unless you are acting as an employee on behalf of your employer or a postgraduate student of an affiliated institution which is paying any applicable article publishing charge ("APC") for Open Access articles. Where the Submitting Author wishes to make the Work available on an Open Access basis (and intends to pay the relevant APC), the terms of reuse of such Open Access shall be governed by a Creative Commons licence – details of these licences and which Creative Commons licence will apply to this Work are set out in our licence referred to above.

Other than as permitted in any relevant BMJ Author's Self Archiving Policies, I confirm this Work has not been accepted for publication elsewhere, is not being considered for publication elsewhere and does not duplicate material already published. I confirm all authors consent to publication of this Work and authorise the granting of this licence.

For peer review only

'I actually felt like I was a researcher myself.'
On Involving Children in the Analysis of Qualitative Paediatric Research in the Netherlands

Malou Luchtenberg, Els Maeckelberghe, Eduard Verhagen
University of Groningen, University Medical Center Groningen

Malou L. Luchtenberg
Beatrix Children's Hospital
University of Groningen, University Medical Center Groningen, the Netherlands

Els L.M. Maeckelberghe
Institute for Medical Education
University of Groningen, University Medical Center Groningen, the Netherlands

A.A. Eduard Verhagen
Beatrix Children's Hospital
University of Groningen, University Medical Center Groningen, the Netherlands

Corresponding author:
Malou L. Luchtenberg
PO Box 30 001, 9700 RB Groningen
The Netherlands
m.l.luchtenberg@umcg.nl
+31 6 25 64 96 59

Word count: 4304 (excl. title page, abstract, references, tables and quotes)

Keywords: involvement, children, paediatrics, patient participation, biomedical research, qualitative research

For peer review only
For peer review only - http://bmjopen.bmj.com/site/about/guidelines.xhtml

ABSTRACT

Objectives
To evaluate the feasibility of a new approach to paediatric research whereby we involved children in analysing qualitative data, and to reflect on the involvement process.

Setting
This was a single-centre, qualitative study in the Netherlands. It consisted of research meetings with individual children at home (Phase 1) or group meetings at school (Phase 2). In Phase 1 we identified themes from a video interview during five one-on-one meetings between a child co-researcher and the adult researcher. In Phase 2, during two group meetings, we explored the themes in detail using fragments from 16 interviews.

Participants
We involved 14 school children (aged 10 to 14) as co-researchers to analyse children's interviews about their experience while participating in medical research. Notes were taken, and children provided feedback. A thematic analysis was performed using a framework approach.

Results
All co-researchers identified themes. The time needed to complete the task varied, as did the extent to which the meetings needed to be structured to improve concentration. The children rated time investment as adequate and they considered acting as co-researcher interesting and fun, adding that they had learnt new skills and gained new knowledge. The experience also led them to reflect on health matters in their own lives. The adult researchers considered the process relatively time intensive, but the project did result in a more critical assessment of their own work.

Conclusion
The new, two-phase approach of involving children to help analyse qualitative data is a feasible research method. The novelty lies in involving children to help identify themes from original interview data, thereby limiting preselection of data by adults, before exploring these themes in detail. Videos make it easier for children to understand the data and to empathise with the interviewees, and limits time investment.

STRENGTHS AND LIMITATIONS OF THIS STUDY

- This study describes a new approach to paediatric research whereby children are involved in analysing qualitative interview data.
- The novelty of this study lies in the fact that children are involved in helping to identify themes from original data as well as exploring the themes in more detail.
- This study explores the use of videos rather than transcripts to present the interviews to relatively young co-researchers.
- The study reflects on children's involvement as co-researchers from the perspective of the children themselves and from that of the adult researchers.
- A limitation of this study is that in test phase presented here we limited the number of child co-researchers and selected the interviews from a larger dataset to include as much variation as possible.

For peer review only

INTRODUCTION

Researchers should be wary of interpretation bias when analysing data of qualitative studies. Qualitative research includes a subjective component that calls for reflexivity.[1] Because the life experiences and social situations of children differ from those of adults, their interpretations of data derived from interviews with children may differ from adults' interpretations. It is therefore desirable to involve children to strengthen the analysis of such qualitative data. There is little evidence in the literature on how children could be effectively involved in scientific data analysis.[2–9] Other challenges regarding patient and public involvement (PPI) [10] of children include lack of funding and lack of time, gatekeeping or power imbalances, and concerns about obtaining knowledge and training on how to involve children.[2,5,11–17] Measuring the impact of a PPI process on research output is difficult because the involvement process itself is complex and therefore its impact cannot by fully captured by evaluating outcomes.[18]

Best and colleagues recently introduced a new method called participatory theme elicitation, whereby a youth advisory panel is involved in qualitative data analysis. The method involves capacity building (training), data selection by adult researchers, data sorting by youth members, and final grouping and analysis by adult researchers.[19] A disadvantage of the method is that it involves data preselection by adults. We hypothesise that it is feasible to involve children from the beginning of data analysis, starting with the identification of themes from original data.

In a larger unpublished study, we collected the experiences of young people regarding their participation in medical research in order to provide recommendations for improving children's participation in research. In the present study, we aimed to explore whether it is feasible to involve children in the analysis of the qualitative data. We designed a two-phase approach that would be effective and efficient: effective in the sense that it involved children to identify themes as well as to explore the themes in more detail and efficient in the sense of limiting time investment. We also aimed to reflect on the involvement process from the perspective of both adults and children. The results of the qualitative data analysis are to be published elsewhere.

For peer review only - http://bmjopen.bmj.com/site/about/guidelines.xhtml

METHODS

This was a single-centre study performed by researchers of University Medical Center Groningen, the Netherlands. We investigated the involvement of children as co-researchers in identifying and analysing themes from interview data presented on video. We conducted the investigation in two phases: Phase 1 consisted of five individual meetings, and Phase 2 of two group meetings. In addition, we reflected on the children's involvement in the analysis process.

Recruitment and sampling

Phase 1

We approached potential co-researchers through national patient support organizations, primary schools, hospitals, social media, and by word-of-mouth. No research experience was required of the participants. Sampling was based on age (9-18 years) and participants were required to be fluent in Dutch because the meetings were held in that language. Initially, seven children volunteered. They each received an information leaflet. Even though recruitment was challenging we managed to achieve our goal of five participants in Phase 1.

Phase 2

We recruited the participants for Phase 2 in collaboration with a specific primary school in Groningen, the Netherlands, where teachers and learners are expected to display an academic mind-set aimed at research and analysis.[20] We invited one class of 15 children of the school's oldest leaners to participate. Ten learners volunteered, one of whom was unable to participate because of illness.

Informed consent

Phase 1

We supplied the potential participants with verbal and written information. We asked them to discuss the study with their parents and to reply by post, e-mail or by telephone. Before the session at the child's home started, he or she read the informed consent form and discussed it with the researcher and a parent. One parent was present throughout the session, but was kindly asked to not interfere with the process. All the children, irrespective of age, were asked to sign the informed consent form to acknowledge that we appreciated their contribution equally. In accordance with the Dutch Medical Research Involving Human Subjects Act, parental consent was obtained in addition to the child's consent.[21] Children were also asked to sign a confidentiality agreement regarding any personal information present in the data

For peer review only - http://bmjopen.bmj.com/site/about/guidelines.xhtml

they analysed. At the end of the session we gave the participants a €10 gift voucher as token of our appreciation of their time and a certificate acknowledging their contribution as co-researchers.

Phase 2

Two adult researchers visited the primary school to meet the learners, their teacher and headmaster, and to introduce the research project. The headmaster agreed to allow the children to participate during school hours. The potential participants received an information leaflet similar to the one given to the participants of Phase 1. We asked them to complete the consent form and the confidentiality agreement at home with a parent, and to return the form to their teacher. At the end of the group meetings we gave the participants a certificate. These children were not given a gift voucher because they participated during school hours.

Data characteristics

Data from the original interview study were analysed by our co-researchers. In addition, the adult researchers took notes during the analyses and written feedback was obtained from the participants before, during, and after participating. In Table 1 we provide additional information on the data collected for the original interview study. Out of a total of 23 interviews two were excluded because they were not videotaped. Another five were excluded because participants and parents had not consented to use the video data.

Table 1. Details of data of the original interview study on children's experiences in medical research

**Study characteristics**	
**Aim**	To explore children's experiences in medical research to obtain recommendations from their perspectives on how to improve children's involvement in research.
**Setting and research team**	Single-centre study conducted by a team of researchers at University Medical Center Groningen, the Netherlands. The research team consisted of an ethicist (EM), paediatrician (EV), and MD/PhD student (ML). All members were trained researchers and/or had previous experience in conducting qualitative research.
**Recruitment and sampling**	Recruitment through health providers from several hospitals, national patient support groups, social media, and by word-of-mouth. Purposive maximum variation sample: children, patients as well as healthy volunteers between 9 and 18 years old who were invited to participate in different types of medical research in the Netherlands and who either took part or declined to take part. The participants had no prior relationships with the members of the research team.
**Informed consent**	Informed consent given by one parent and the child or, in accordance to Dutch law, from 16 years and older by the child only.

For peer review only - http://bmjopen.bmj.com/site/about/guidelines.xhtml

**Data collection**	Twenty three semi-structured, in-depth interviews, lasting between 30 and 100 minutes, with children about their experiences in taking part in medical research, including recommendations for improvement of children's involvement in informed consent procedures and the research itself. A topic guide was developed based on a previous study in the United Kingdom. Interviews performed by ML took place at children's homes and were recorded on audio or video, transcribed verbatim, and returned to the participants. No comments from participants were received. Data collection continued until we reached data saturation of main themes.
**Ethical approval**	The conclusion of the Medical Ethics Review Board of the University Medical Center Groningen was that this study, no. M16.192386, 10 May 2016 fell beyond the scope of the Dutch Medical Research Involving Human Subjects Act .

The co-researchers received a brief interactive introduction to paediatric research and to the original interview study. They were asked to identify the main topics in video interviews and to summarise them on mind maps *(A type of diagram with lines and circles for organising information so that it is easier to use or remember*).[22, p1] To prevent the children from becoming 'little adult researchers' we did not train them extensively. Extensive training would also have been more time consuming for both the children and the researchers.

In Phase 1 the five co-researchers collaborated with the coordinating researcher (PPIA1 – PPI adult researcher 1) in a one-on-one session to identify the main themes in five different interviews. Together they watched a video of between 25 to 45 minutes of another young person and discussed the emerging themes. This took place at the participants' home and lasted between two and three and a half hours. During Phase 1 the adult researcher spent a total of eleven hours travelling to and from co-researchers homes.

During the two group meetings in Phase 2, we explored in detail two themes that had been identified during Phase 1. For this purpose we compiled two five-minute videos from fragments of several interviews from the dataset of the original study. The group meetings took place at a local primary school and lasted approximately two and a half hours. Travelling time for adult researchers was two hours, including the introductory meeting when we handed out the information sheets. In addition to travelling time, the time investment of adult researchers was approximately five hours. This included the time spent preparing the instructions and making the compilation videos. The material costs of the project were low and the travelling costs minimal.

For peer review only - http://bmjopen.bmj.com/site/about/guidelines.xhtml

The aim of the analysis process in both phases was to identify the main themes in the video through open, unstructured discussions with the child co-researchers. To facilitate interaction and discussion the co-researchers and the adult researchers could pause the video at any time. They took notes of what they thought the interviewee found important. PPIA1 and PPIA2 allowed moments of personal reflection, but were on guard for potential intertwining of co-researchers' personal experiences with interviewees' experiences. After watching the video the co-researchers drew a mind map depicting their interpretation of the connection between different themes.[22] The mind maps were drawn on A3-sized sheets of paper, using different sizes of sticky notes, and coloured pens. Besides, the participants were free to use materials of their own choice. The researcher asked the participants questions about the importance of certain themes, the identification of overlapping themes, and the reason why they had chosen a certain theme. The participants led the discussion and made the final decision in case of a disagreement about a theme.

Data collection and analysis

Before the study actually commenced, we asked the child co-researchers why they would like to take part. The adult researchers took notes of how participants fulfilled their role as co-researcher, and how the participants reflected on this process. Child co-researchers completed a feedback form after the analysis (Table 2). In addition, we briefly evaluated the process orally. All meetings were recorded on audio tape.

We performed a thematic analysis using a framework approach.[23] Familiarisation and initial theme identification was done by PPIA1 (ML) and discussed with EM (individual meetings) and PPIA2 (group meetings) based on the audio tape, notes, and the participants' written feedback. Some themes, such as time investment, were identified in advance from the literature, others were derived from the data. Themes were refined and conceptualised during regular meetings with the research team and any disagreements were discussed and a final decision reached by consensus. Because the sample for this exploratory study was relatively small, we did not aim for data saturation.

Table 2. Feedback form

1. Did you understand beforehand what your role was in the project? (No / a little / yes)<br>2. How could we improve the information about working as a co-researcher?	6. Would you recommend other children to become co-researchers? Why?<br>7. How would you rate your time investment? (Too long, adequate, too short)

For peer review only - http://bmjopen.bmj.com/site/about/guidelines.xhtml

3. What was it like for you to work as a co-researcher?<br>a. positive aspects<br>b. points of improvement<br>4. Did you learn anything from being a co-researcher? If so, what did you learn?<br>5. Would you like to be a co-researcher more often?	8. a. What did you think of the EUR 10 gift voucher? (Phase 1)<br>b. What did you think about participating in this project during school hours? Why? (Phase 2)<br>9. Do you have suggestions for improving this evaluation form?

Patient and public involvement

We explored the feasibility of an approach whereby children were involved in qualitative data analysis, both in helping adult researchers to identify themes from original data as in exploring the themes in more detail.

RESULTS

Participant characteristics

Fourteen children, eight girls and six boys, participated as co-researchers in this study. Two participants had experience because they had been chronic patients themselves. None of the children had been a co-researcher before. Tables 3 and 4 show the characteristics of the child and adult participants.

Table 3. Child participant characteristics

Characteristics of child co-researchers	N* (%)**
Sex	
Girl	8 (57)
Boy	6 (43)
Age	
10 years	1 (7)
11 years	10 (71)
12 years	2 (14)
13 years	0 (0)
14 years	1 (7)
School attended	
Primary School	13 (93)
Secondary School	1 (7)
Hospital/Disease experience (lived experience)	
Currently suffering from a disease	2 (14)
Hospitalization or minor surgery in the past	6 (43)
Family member(s) who suffer from a disease	2 (14)
None	4 (29)
Research experience as participant	
Yes	2 (14)
No	12 (86)
Experience as co-researcher	
Yes	0 (0)
No	14 (100)

For peer review only

For peer review only - http://bmjopen.bmj.com/site/about/guidelines.xhtml

* Number of child participants; N = 14
** Rounded to nearest whole number

Table 4. Adult participants characteristics

Participant	Age	Sex	Experience illness /hospital	Research experience (participating in research)	Research experience (performing research)
PPIA1	27	Woman	As a medical student. Graduated as medical doctor in August 2019.	Yes. Participant in two large cohort studies for several years.	Training and experience in qualitative research for PhD.
PPIA2	23	Woman	As a medical student. Started internships in September 2019.	No previous experience in medical research participation.	Trained in qualitative research as a former psychology student.

Reflection and evaluation of the involvement process

The results can be divided into five main themes: (1) understanding the study procedures, (2) empowerment, (3) reflection on health and illness, (4) interest in the bigger picture, and (5) reflection on time investment. The results of the feedback form are summarised in Table 5.

Table 5. Summary of written feedback

Theme (question from Table 2)	Summary of written feedback	N* (%)**
**Understanding the study procedures** (1,2,9)	*Understanding their role as co-researcher before start****	
	No	0 (0)
	A little	12 (86)
	Yes	2 (14)
	Suggestions for improving the information about working as co-researcher	
	Don't know	
	Everything was clear	4 (29)
	Use fewer difficult words	5 (36)
	Explain that we had to take notes and create a mind map	4 (29)
		1 (7)
	Suggestions for improving the feedback form of the co-researcher project	
	Adding a question about the overall experience	1 (7)
	No recommendations	13 (93)
**Empowerment** (3-6)	*Positive experience as co-researcher*	
	Fun	14 (100)
	Interesting	4 (29)
	Helping other children	1 (7)
	Learning something new	1 (7)
	Time investment was okay	2 (14)
	Receiving a certificate	1 (7)
	Points of improvement for co-researcher project	
	No points of improvement	12 (86)
	Shorter interviews	1 (7)
	The project should take the whole school day (instead of a half one)	1 (7)
	Lessons learnt from being a co-researcher	
	Taking notes	2 (14)
	Critical thinking and listening	4 (29)

For peer review only

	About a medical condition	2 (14)
	About doing research	2 (14)
	About how children think and feel about research	2 (14)
	That children think differently from adults	1 (7)
	That it is fun and that you learn a lot	1 (7)
	Not really	1 (7)
	Would like to be co-researcher more often including reason	
	Yes, because it's fun	12 (86)
	Yes, because it's interesting	5 (36)
	Yes, because I like to help people	1 (7)
	Yes, I know what to expect now	1 (7)
	Yes, if it doesn't hurt	1 (7)
	It is fun, but depends on how much time I have	1 (7)
	Would you recommend others to become co-researcher?	
	Yes, because it's (super) fun	9 (64)
	Yes, because it's interesting/you learn something from it	7 (50)
	Yes, because you receive a gift voucher	1 (7)
	Yes, because you can help other people	2 (14)
	Yes, because you get sweets	1 (7)
	Yes, but it depends on whether it suits them	1 (7)
**Time investment** (7,8b)	*Rating of time investment****	
	Too long	1 (7)
	Adequate	12 (86)
	Too short	1 (7)
	*Thoughts on having this project during school time (Phase 2, n=9)*****	
	Fun/good, because you didn't have to do schoolwork	6 (67)
	Fun/good, because you don't miss free time after school	4 (44)
	Don't mind	1 (11)
**Compensation** (8b)	*Thoughts on receiving a gift voucher (Phase 1, n=5)*****	
	Fun/good	5 (100)
	Not necessary	2 (40)
	Creative	1 (20)

* Number of child participants; N = 14. Some participants provided more than one answer
** Rounded to nearest whole number
*** The feedback provided is based on a multiple choice question
**** Calculation based on a sub-selection of total participants, because children took part in a different phase

For peer review only

1. Understanding of study procedures

When we asked the participants in the group meetings what they expected of the session, they remembered that the main idea was '*doing research about research.*' Nonetheless, not everyone remembered the details such as whether photographs would be taken. Even though the information was especially written with children in mind, the participants recalled that it was mostly read and signed by their parents, and had not always been discussed with them. In the case of the co-researchers in the individual meetings this was different. Here the researcher and the parents were present and encouraged the children to complete the form themselves. Parents were available in the background in case their help was needed.
Most children reported that they had a general idea about what their role was in the project. We explained their role to them in detail during the actual project. One of them explained:

For peer review only - http://bmjopen.bmj.com/site/about/guidelines.xhtml

'I already understood it but once you are doing it you understand it [better].' (Girl, age 10-11 years old, individual meeting)

2. Empowerment

By involving children as co-researchers they gained knowledge, they learnt new skills, and they became more confident in fulfilling their role during the analysis process. This was mostly because they enjoyed the new experience of contributing to research or helping others. Children felt empowered by working as a co-researcher. One of them explained it as:

'It actually felt a bit like I was a researcher myself.' (Girl, age 10-11 years old, group meeting)

They reported gaining new knowledge about certain health matters and they realised what it is like to be ill. They learnt how to do research, how to think critically, and how to take notes. Here are some representative answers given:

'You have to think carefully before you draw conclusions.' (Boy, age 10-11 years old, group meeting)

'A bit about how ill children felt afterwards [after participating in research].' (Girl, age 10-11 years old, group meeting)

All the participants were positive about the idea of being a co-researcher more often, mostly for similar reasons as for wanting to take part in the first place:

'Yes, it was fun, relaxed, and instructive.' (Girl, age 10-11 years old, group meeting)

One participant, the only one attending secondary school, mentioned it would depend on whether he had the time to take part because of homework and sports activities in his free time. All participants reported that they would recommend others to become co-researchers, though one participant acknowledged that it might not suit everyone. She mentioned that some children might not enjoy it or might not have the skills to do such work.

For peer review only

During the analysis process in Phase 1, the co-researchers grew noticeably more confident as time progressed. The adult researcher retreated to the background and stimulated the co-researchers to take the lead, which most of them did eventually. One participants actually asked the researcher questions about her observations, instead of the other way around. Most participants, however, needed some form of structuring support from the adult researcher throughout.

During the group meetings the support needed from the adult researcher was different. The co-researchers needed more structuring because of group dynamics. Confusion due to competing voices ensued, and shyer children tended to not be heard. Interestingly, the two groups chose different ways of translating their notes to the mind map. In one group the adult researcher noticed a clear distinction in participants' role preferences, and the co-researchers divided the roles between themselves. Some preferred an executive role, such as writing down themes on the 'sticky notes', while others preferred to simply express their ideas and to play a more coordinating role. Some found it difficult to summarise their notes and suggested first underlining important notes:

> *'We could also just first underline what we think is important.'* (Girl, age 10-11 years old, group meeting)

The co-researchers in the other group together decided that they all wanted to write down their own notes on 'sticky notes' and to put them all on the mind map. The outcome was a mind map that displayed different topics as well as providing insight into how important the individuals thought a certain topic was by the number of 'sticky notes' on the same topic. Others only needed a bit more time and space to find their own role.

It was challenging for the adult researchers to not provide answers themselves when the participants indicated that they did not know how to proceed with the analysis. By repeating or rephrasing their question and by acknowledging that they were doing the right thing, the adult researcher could reinforce the children. Both adult researchers were surprised by the co-researcher's achievements. Throughout the project the co-researchers displayed the ability to identify themes and to visualise them in mind maps, underlining the feasibility of this approach and its value for interpreting data.

For peer review only - http://bmjopen.bmj.com/site/about/guidelines.xhtml

The researchers learnt a great deal from involving children in the analyses because the participants were very open and direct in their feedback. If they did not understand something, for example, if a question was not clearly formulated, or if they did not understand medical jargon such as ‘treatment protocol’, they said so immediately.

Whereas the adult researchers tended to generalise findings, the co-researchers stuck more to the original data. One topic, for example, dealt with recommendations to researchers for improving young people’s experiences when participation in medical research. The interviewees mentioned issues like making hospital visits more enjoyable and gave concrete suggestions. All the researchers, adults and children alike, started from the original data, but there appeared to be a difference in analysis. The concrete suggestions in the children’s analyses remained and made their recommendations were therefore emphasised, while the adults generalised them into ‘ways to brighten up the visits’. The co-researchers brought the adults back to the basics that were important to children.

3. Reflection on health and illness

The participants empathised with the young people who shared their experiences in the video. They wondered whether they were still ill or asked if they still lived and hoped they were all right. The following is a representative example:

> *‘None of these children [in the video] is deadly ill, right?’* (Girl, age 10-11 years old, group meeting)

The adult researcher explained the different illnesses the interviewees had, and mentioned that some of them had been critically ill, for example, with leukaemia, but that they were stable at the time they were interviewed.

The co-researchers asked questions about the illnesses and what the consequences might be for the lives of the children such as two siblings with a hereditary condition. They also shared their own experiences, for example, about relatives who had cancer.

4. Interest in the bigger picture

Even though we only involved the participants in the analysis stage of the main interview study, they were well aware that this was part of a bigger study. They asked about the other

For peer review only - http://bmjopen.bmj.com/site/about/guidelines.xhtml

participants and previous experiences with involving children in research analysis, and wondered whether we would show the mind maps they had made to the interviewees. Some of the questions they asked were:

> *'Have you been to children's homes?' 'Are you also doing this [project] at other schools?' 'How many times have you done this?'* (Several co-researchers, boys and girls, age 10-14 years old, group meetings, not clearly identifiable from the audio)

Hearing that they were the first group of children that participated as co-researchers in this project made them feel special. They expressed the wish to receive the final results and hoped we would do this project again. The co-researchers had a broader interest than just fulfilling their role as co-researchers. They also asked personal questions, such as why the adult researchers did this research project and whether it was part of their university training. They also acknowledged and enjoyed helping the adult researchers with their research:

> *'It is, of course, good for you [adult researchers] that we participate so that you can continue doing research about research of the research.'* (Girl, 10-11 years old, group meeting)

5. Reflection on time investment

Most children said the time investment was appropriate. One child in the group meetings reported that a shorter meeting would have been better because some children became distracted:

> '*Because at the end we were chatting a bit, [we got] distracted.'* (Boy, age10-11 years old, group meeting)

This was also observed by the adult researchers. Another participant, however, reported that he would have like the session to last longer because he really liked it and did not want to return to his normal schoolwork. The Phase 2 participants all reported that it was an interesting and fun alternative to normal school tasks. They thought it was good to do the project during school time because this way they would not miss out on any free time. One of the participants explained this:

For peer review only - http://bmjopen.bmj.com/site/about/guidelines.xhtml

'It was fun because you didn't have to work, and if it hadn't been during school it was inconvenient.' (Girl, age 10-11 years old, group meeting)

From the adult perspective, time was invested in recruitment, developing material to introduce and explain the procedure, and thinking about how to best involve children. The actual analysis with co-researchers was time-intensive and lasted longer than we had expected. Nevertheless, the time invested was considered reasonable given the empowerment of children, their learning new skills, and their views on our data provided additional insight, which is promising for the interpretation of our interviews.

DISCUSSION

Little evidence is available on how to involve children in research.[11] In this paper we describe how we involved relatively young children in the analysis of medical research interviews analysis using a two-phase approach. We deployed various strategies to avoid a tokenistic approach, to address challenges regarding time management, and to empower children during the process.

Two-phase approach

Involving relatively young children, aged 10 to14, in our interview analyses was a challenging process. Our aim was to limit preselection of data by adults by introducing one-on-one meetings during which research interviews were analysed by young co-researchers. This approach could be considered an extension of Best's 'participatory theme elicitation' method.[19] Even though the sessions lasted longer than originally intended, the fact that we could focus on one individual worked to our advantage. In our opinion the project benefitted from the fact that the individual meetings were held at the children's homes, which constituted a safe and familiar environment. This confirms findings from Dovey-Pearce and colleagues who highlighted the importance of face-to-face meetings to establish relationships.[18] The themes identified during Phase 1 were explored in detail during group meetings in Phase 2. Working with a number of co-researchers in this phase improved the rigor of the qualitative analysis. In addition, there was an unexpected positive result for the co-researchers who were classmates. Their bond was strengthened by their shared research experience and by reflecting on health and illness together. The two-phase approach enabled us to achieve our research goals and to empower our co-researchers, while keeping within reasonable the time limits. This applied to adult and child researchers alike.

For peer review only - http://bmjopen.bmj.com/site/about/guidelines.xhtml

Use of videos in analysis

Our aim was to involve child co-researchers in interview analyses in an effective and efficient way. Data analysis in qualitative research is often lengthy process involving large quantities of text. Locock and colleagues reported that young people reading through the transcripts was tedious and inefficient. They concluded that it was more effective to discuss the data rather than digging into detailed transcripts.[9] We decided to explore other ways of involving young co-researchers in interview analyses. Visuals such as photographs, drawings, or mapping methods are often used in collaborations with young children to collect data about children's views on, for example, what they value in their lives.[24] Darbyshire and colleagues reported that using a variety of qualitative visual techniques is helpful for engaging children in research. It also provides a good way for children to express their views. There is, however, a problem with using visuals as a participatory method rather than in analysis, as Darbyshire and colleagues pointed out: *'…having children take photographs and then having only adults "interpret" (or possibly misinterpret) them is potentially an adultist approach to research on children that we sought to avoid.'*[25] For this reason we used videos in the analysis stage to visualise the interview data to be analysed. Our study confirmed the benefits expressed by Darbyshire and colleagues. The co-researchers enjoyed the creative process of developing the mind map and the videos helped them understand and empathise with the interviewees. Using videos rather than transcripts made the process more time-efficient, while preserving the effectiveness of a thematic analysis. Another benefit of videos over transcripts concerned the rigor of the qualitative data analyses. The analysis of interview data is often assumed to start as soon as the interview has been fully transcribed but, even in case of a verbatim transcript including descriptions of vocal emotions such as laughter, the loss of key elements, such as volume of voices and facial expressions, remains. This could present interviewees' experiences in a more abstract way than the original data show.[26, 27] Put differently, by using videos we possibly started the analysis with a more authentic representation of the data.

Additional considerations and further research

Time investment is an important consideration when developing ways of involving children in research analysis. In the method developed by Best and colleagues the analysis is limited to two hours. An additional time investment of four times 90 to 120 minutes is asked of participants for 'capacity building.' In these sessions, young people learn how to design and

For peer review only - http://bmjopen.bmj.com/site/about/guidelines.xhtml

conduct a study, how to perform qualitative data analyses, and they receive an introduction into the subject matter of their data.[19] We purposely did not train our co-researchers to avoid shaping them to comply with our idea of a qualitative researcher, and to limit time investment. Though we cannot make a comparison, the little training we gave our co-researchers did not seem to have had a negative impact on the result.

In addition to time investment, timing of research meetings should be considered. Many Young People's Advisory Groups generally plan their meetings during school holidays or weekends.[28] The INVOLVE Advisory Group of the United Kingdom's National Institute for Health Research, which supports active public involvement in the National Health Service, public health, and social care research,[29] identified parents and schools as a significant barrier to public involvement during school hours: *…lack of schools' recognition of the value of their work sometimes acts as a barrier to them attending events which involve travel in school hours.*[13, p12] Nevertheless, thanks to a cooperative headmaster, we managed to set up a collaboration with a primary school for Phase 2 of our study, and planned the group meetings during school hours. For our co-researchers in the group meetings, participating during school time was preferable to after school, because they reported that they had busy schedules or had to do homework during their free time.

Our results showed that children tend to include more concrete topics in their analyses, whereas adults analyse data in a more abstract way. This is in line with the cognitive development of children, who transform from concrete to abstract conceptualisation later in adolescence.[30, 31] Consequently, we expect that an evaluation of the data analysis process performed by children, young people, and adults will provide additional interesting insights. Recently, we started testing our two-phase approach with young people aged 16 to 18. They will use this project for a school assignment, thereby creating a situation that is mutually beneficial. If this proves successful, we consider setting up a long-term collaboration with primary schools and secondary schools to optimise collaboration between researchers and children to help decrease the knowledge gap between academia and society.

Study limitations

To test this new approach we started with a small group of young co-researchers. As a consequence, we had to select of interviews from a larger dataset to analyse in Phase 1. We aimed for as much variation as possible within this selection, but we also needed to be

For peer review only - http://bmjopen.bmj.com/site/about/guidelines.xhtml

pragmatic, regarding the length of the interviews for instance. In addition, in our method children were not involved in making choices about specific quotes used in the results sections. As recruiting co-researchers was challenging, sampling was limited to age and to fluency in the Dutch language. Maximum variation sampling would, however, be preferable to improve reflexivity.

CONCLUSION

We suggest that the two-phase approach to involving young children in analysing qualitative data is feasible. Its novelty lies in recruiting children to help identify themes from original data before the themes are explored in detail. Thus preselection of data by adults is limited. By combining one-on-one meetings and group meetings the two-phase approach is an effective and efficient way of involving relatively young children in analysing qualitative data. Additional benefits are that children reflect on health and illness in their own lives, they are empowered, and engaged in medical research. We recommend presenting the interview data on videos rather than through transcripts. Videos make it easier for children to understand the data, to empathise with the interviewees, and it limits time investment.

ETHICS APPROVAL

The Medical Ethics Review Board of the University Medical Center Groningen concluded that neither this study (M18.2334032, 24 July 2018) nor our larger study (M16.192386, 10 May 2016) fall within the scope of the Dutch Medical Research Involving Human Subjects Act.

ACKNOWLEDGEMENTS

We thank all the participants in the original interview study and our co-researcher participants in this project. Because this is a meta-analysis of the PPI project, we decided not to invite the co-researchers as co-author of this article. We thank Laura Postma (PPIA2) for helping to facilitate the group meetings.

FUNDING STATEMENT

The PPI study itself was a low-cost project. Data collection for the main interview study was funded by the University of Groningen as part of the MD/PhD program of Malou Luchtenberg. This funding body has no role in the design of the study and collection, analysis, or interpretation of data, or in writing the manuscript.

COMPETING INTERESTS STATEMENT

None declared.

DATA SHARING STATEMENT

The original dataset with audiotaped and videotaped interviews is not available for the public on account of privacy concerns.

For peer review only - http://bmjopen.bmj.com/site/about/guidelines.xhtml

AUTHOR'S CONTRIBUTION

ML, EM and EV designed the original study. ML was responsible for the data collection in Phase 1 and the group meetings were led by ML (PPIA1). Analysis was performed by ML, checked by EM, and discussed with EV. ML made the first draft of the article, which was subsequently discussed within the team and reviewed by EM and EV. All authors read and approved the final manuscript.

REFERENCES

1. Hennink M, Hutter I, Bailey A. The nature of qualitative research. Subjectivity and the need for reflexivity. In: Qualitative Research Methods. London: Sage 2011:19-23.
2. Taylor RM, Whelan JS, Gibson F, et al. Involving young people in BRIGHTLIGHT from study inception to secondary data analysis: insights from 10 years of user involvement. *Res Involv Engagem* 2018;4(1):1–14. https://doi.org/10.1186/s40900-018-0135-x
3. Jamal F, Langford R, Daniels P, et al. Consulting with young people to inform systematic reviews: an example from a review on the effects of schools on health. *Heal Expect* 2015;18(6):3225–35. https://doi.org/10.1111/hex.12312
4. Coad J, Evans R. Reflections on practical approaches to involving children and young people in the data analysis process. *Child Soc* 2008 Jan;22(1):41–52. https://doi.org/10.1111/j.1099-0860.2006.00062.x
5. Clavering EK, McLaughlin J. Children's participation in health research: from objects to agents? *Child Care Health Dev* 2010;36(5):603–11. https://doi.org/10.1111/j.1365-2214.2010.01094.x
6. Brady L-M, Preston J. Evaluating the extent and impact of young people's involvement in National Institute for Health Research (NIHR) studies: an assessment of feasibility. 2017 April. http://www.invo.org.uk/wp-content/uploads/2017/06/Impact-of-CYPs-involvement_JLI__GenR.pdf (accessed 01-07-2019)
7. Lundy L, McEvoy L, Byrne B. Working with young children as co-researchers: an approach informed by the United Nations Convention on the Rights of the Child. *Early Educ Dev* 2011;22(5):714–36. https://doi.org/10.1080/10409289.2011.596463
8. Holland S, Renold E, Ross NJ, et al. Power, agency and participatory agendas: a critical exploration of young people's engagement in participative qualitative research. *Childhood* 2010;17(3):360–75. https://doi.org/10.1177/0907568210369310
9. Locock L, Kirkpatrick S, Brading L, et al. Involving service users in the qualitative analysis of patient narratives to support healthcare quality improvement. *Res Involv Engagem* 2019;5(1):1–11. https://doi.org/10.1186/s40900-018-0133-z
10. INVOLVE. What is public involvement in research? *NHS Natl Inst Heal Res* 2015. http://www.invo.org.uk/find-out-more/what-is-public-involvement-in-research-2/ (accessed 10-07-2019)
11. Bird D, Culley L, Lakhanpaul M. Why collaborate with children in health research: an analysis of the risks and benefits of collaboration with children. *Arch Dis Child - Educ Pract Ed* 2013;98(2):42–8. https://doi.org/10.1136/archdischild-2012-303470
12. Preston J, Stones SR, Davies H, et al. How to involve children and young people in what is, after all, their research. *Arch Dis Child* 2019;104(5):494–500. https://doi.org/10.1136/archdischild-2018-315118
13. Sonpal K, Walker E, Swallow V, et al. Report on involving children and young people in research. Southampton: INVOLVE. 2019. https://www.invo.org.uk/wp-content/uploads/2019/06/77620-YPv3-Booklet-WEB.pdf (accessed 01-07-2019)

For peer review only - http://bmjopen.bmj.com/site/about/guidelines.xhtml

14. Blackburn S, McLachlan S, Jowett S, et al. The extent, quality and impact of patient and public involvement in primary care research: a mixed methods study. *Res Involv Engagem* 2018;4(1):1–18. https://doi.org/10.1186/s40900-018-0100-8
15. Domecq JP, Prutsky G, Elraiyah T, et al. Patient engagement in research: a systematic review. *BMC Heal Serv Res* 2014;14(89):1–9. https://doi.org/10.1186/1472-6963-14-89
16. Kellett M, Forrest R, Dent N, et al. "Just teach us the skills please, we'll do the rest": Empowering ten-year-olds as active researchers. *Child Soc* 2004;18(5):329–43. https://doi.org/10.1002/chi.807
17. Chappell P, Rule P, Dlamini M, et al. Troubling power dynamics: youth with disabilities as co-researchers in sexuality research in South Africa. *Childhood* 2014;21(3):385–99. https://doi.org/10.1177/0907568214525427
18. Dovey-Pearse G, Walker S, Fairgrieve S, et al. The burden of proof: The process of involving young people in research. *Helath Expectations* 2019;22:465-474. https://doi.org/10.1111/hex.12870
19. Best P, Badham J, Corepal R, et al. Network methods to support user involvement in qualitative data analyses: an introduction to Participatory Theme Elicitation. *Trials* 2017;18(1):1–14. https://doi.org/10.1186/s13063-017-2289-5
20. Brederoschool. Academische Basisschool 2019. Groningen, the Netherlands. https://brederoschool.openbaaronderwijsgroningen.nl/academischebasisschool/index.html (accessed 09-11-2018)
21. Dutch Medical Research Involving Human Subjects Act (WMO), 26 February 1998. https://wetten.overheid.nl/BWBR0009408/2019-04-02 (accessed 09-04-2019)
22. Cambridge University Press. Meaning of mind map in English. Cambridge Dictionary. 2019. https://dictionary.cambridge.org/dictionary/english/mind-map (accessed 19-09-2019)
23. Smith J, Firth J. Qualitative data analysis: the framework approach. Nurse Researcher 2011;18(2):52–62. https://doi.org/10.7748/nr2011.01.18.2.52.c8284
24. Coad J. Using art-based techniques in engaging children and young people in health care consultations and/or research. *J Res Nurs* 2007;12(5):487–97. https://doi.org/10.1177/1744987107081250
25. Darbyshire P, Macdougall C, Schiller W. Multiple methods in qualitative research with children: more insight or just more? *Qual Res* 2005;5(4):417–436. https://doi.org/10.1177/1468794105056921
26. Hewitt J. Ethical components of researcher-researched relationships in qualitative interviewing. *Qual Health Res* 2007;17(8):1149–59. https://doi.org/10.1177/1049732307308305
27. Alldred P, Gillies V. Eliciting research accounts: re/producing modern subjects. Ethics in Qualitative Research. London: Sage 2002:146–65.
28. Gaillard S, Malik S, Preston J, et al. Involving children and young people in clinical research through the forum of a European Young Persons' Advisory Group: needs and challenges. *Fundam Clin Pharmacol* 2018 Aug;32(4):357–62. https://doi.org/10.1111/fcp.12360
29. National Institute for Health Research. About INVOLVE. https://www.invo.org.uk/about-involve/ (accessed 04-03-2020)
30. Garrison W, Felice ME. Adolescence. In: Carey WB, Crocker AC, Coleman WL, Elias ER, Feldman HM, eds. Developmental-Behavioral Pediatrics (Fourth Edition) 2009:62–73. https://doi.org/https://doi.org/10.1016/B978-1-4160-3370-7.00006-7
31. Christie D, Viner R. Adolescent development. *BMJ (Clinical Research Ed.)* 2005;330:301. https://doi.org/10.1136/bmj.330.7486.301

For peer review only - http://bmjopen.bmj.com/site/about/guidelines.xhtml

For peer review only

For peer review only - http://bmjopen.bmj.com/site/about/guidelines.xhtml

BMJ Open

'I actually felt like I was a researcher myself.' On Involving Children in the Analysis of Qualitative Paediatric Research in the Netherlands

Journal:	*BMJ Open*
Manuscript ID	bmjopen-2019-034433.R4
Article Type:	Original research
Date Submitted by the Author:	21-Jul-2020
Complete List of Authors:	Luchtenberg, Malou; University of Groningen; University Medical Center Groningen, Department of Pediatrics<br>Maeckelberghe, Els; University of Groningen, Institute for Medical Education; University Medical Center Groningen<br>Verhagen, Eduard; University of Groningen; University Medical Center Groningen , Department of Pediatrics
**Primary Subject Heading**:	Paediatrics
Secondary Subject Heading:	Ethics, Qualitative research, Research methods
Keywords:	Involvement, Children, PAEDIATRICS, Patient Participation, Biomedical Research, QUALITATIVE RESEARCH

SCHOLARONE™
Manuscripts

I, the Submitting Author has the right to grant and does grant on behalf of all authors of the Work (as defined in the below author licence), an exclusive licence and/or a non-exclusive licence for contributions from authors who are: i) UK Crown employees; ii) where BMJ has agreed a CC-BY licence shall apply, and/or iii) in accordance with the terms applicable for US Federal Government officers or employees acting as part of their official duties; on a worldwide, perpetual, irrevocable, royalty-free basis to BMJ Publishing Group Ltd ("BMJ") its licensees and where the relevant Journal is co-owned by BMJ to the co-owners of the Journal, to publish the Work in this journal and any other BMJ products and to exploit all rights, as set out in our licence.

The Submitting Author accepts and understands that any supply made under these terms is made by BMJ to the Submitting Author unless you are acting as an employee on behalf of your employer or a postgraduate student of an affiliated institution which is paying any applicable article publishing charge ("APC") for Open Access articles. Where the Submitting Author wishes to make the Work available on an Open Access basis (and intends to pay the relevant APC), the terms of reuse of such Open Access shall be governed by a Creative Commons licence – details of these licences and which Creative Commons licence will apply to this Work are set out in our licence referred to above.

Other than as permitted in any relevant BMJ Author's Self Archiving Policies, I confirm this Work has not been accepted for publication elsewhere, is not being considered for publication elsewhere and does not duplicate material already published. I confirm all authors consent to publication of this Work and authorise the granting of this licence.

For peer review only

'I actually felt like I was a researcher myself.'

On Involving Children in the Analysis of Qualitative Paediatric Research in the Netherlands

Malou Luchtenberg, Els Maeckelberghe, Eduard Verhagen

University of Groningen, University Medical Center Groningen

Malou L. Luchtenberg

Beatrix Children's Hospital

University of Groningen, University Medical Center Groningen, the Netherlands

Els L.M. Maeckelberghe

Institute for Medical Education

University of Groningen, University Medical Center Groningen, the Netherlands

A.A. Eduard Verhagen

Beatrix Children's Hospital

University of Groningen, University Medical Center Groningen, the Netherlands

Corresponding author:

Malou L. Luchtenberg

PO Box 30 001, 9700 RB Groningen

The Netherlands

m.l.luchtenberg@umcg.nl

+31 6 25 64 96 59

Word count: 4304 (excl. title page, abstract, references, tables and quotes)

Keywords: involvement, children, paediatrics, patient participation, biomedical research, qualitative research

For peer review only - http://bmjopen.bmj.com/site/about/guidelines.xhtml

For peer review only

ABSTRACT

Objectives
To evaluate the feasibility of a new approach to paediatric research whereby we involved children in analysing qualitative data, and to reflect on the involvement process.

Setting
This was a single-centre, qualitative study in the Netherlands. It consisted of research meetings with individual children at home (Phase 1) or group meetings at school (Phase 2). In Phase 1 we identified themes from a video interview during five one-on-one meetings between a child co-researcher and the adult researcher. In Phase 2, during two group meetings, we explored the themes in detail using fragments from 16 interviews.

Participants
We involved 14 school children (aged 10 to 14) as co-researchers to analyse children's interviews about their experience while participating in medical research. Notes were taken, and children provided feedback. A thematic analysis was performed using a framework approach.

Results
All co-researchers identified themes. The time needed to complete the task varied, as did the extent to which the meetings needed to be structured to improve concentration. The children rated time investment as adequate and they considered acting as co-researcher interesting and fun, adding that they had learnt new skills and gained new knowledge. The experience also led them to reflect on health matters in their own lives. The adult researchers considered the process relatively time intensive, but the project did result in a more critical assessment of their own work.

Conclusion
The new, two-phase approach of involving children to help analyse qualitative data is a feasible research method. The novelty lies in involving children to help identify themes from original interview data, thereby limiting preselection of data by adults, before exploring these themes in detail. Videos make it easier for children to understand the data and to empathise with the interviewees, and limits time investment.

STRENGTHS AND LIMITATIONS OF THIS STUDY

- This study describes a new approach to paediatric research whereby children are involved in analysing qualitative interview data.
- The novelty of this study lies in the fact that children are involved in helping to identify themes from original data as well as exploring the themes in more detail.
- This study explores the use of videos rather than transcripts to present the interviews to relatively young co-researchers.
- The study reflects on children's involvement as co-researchers from the perspective of the children themselves and from that of the adult researchers.
- A limitation of this study is that in test phase presented here we limited the number of child co-researchers and selected the interviews from a larger dataset to include as much variation as possible.

For peer review only

INTRODUCTION

Researchers should be wary of interpretation bias when analysing data of qualitative studies. Qualitative research includes a subjective component that calls for reflexivity.[1] Because the life experiences and social situations of children differ from those of adults, their interpretations of data derived from interviews with children may differ from adults' interpretations. It is therefore desirable to involve children to strengthen the analysis of such qualitative data. There is little evidence in the literature on how children could be effectively involved in scientific data analysis.[2–9] Other challenges regarding patient and public involvement (PPI) [10] of children include lack of funding and lack of time, gatekeeping or power imbalances, and concerns about obtaining knowledge and training on how to involve children.[2,5,11–17] Measuring the impact of a PPI process on research output is difficult because the involvement process itself is complex and therefore its impact cannot by fully captured by evaluating outcomes.[18]

Best and colleagues recently introduced a new method called participatory theme elicitation, whereby a youth advisory panel is involved in qualitative data analysis. The method involves capacity building (training), data selection by adult researchers, data sorting by youth members, and final grouping and analysis by adult researchers.[19] A disadvantage of the method is that it involves data preselection by adults. We hypothesise that it is feasible to involve children from the beginning of data analysis, starting with the identification of themes from original data.

In a larger unpublished study, we collected the experiences of young people regarding their participation in medical research in order to provide recommendations for improving children's participation in research. In the present study, we aimed to explore whether it is feasible to involve children in the analysis of the qualitative data. We designed a two-phase approach that would be effective and efficient: effective in the sense that it involved children to identify themes as well as to explore the themes in more detail and efficient in the sense of limiting time investment. We also aimed to reflect on the involvement process from the perspective of both adults and children. The results of the qualitative data analysis are to be published elsewhere.

For peer review only - http://bmjopen.bmj.com/site/about/guidelines.xhtml

METHODS

This was a single-centre study performed by researchers of University Medical Center Groningen, the Netherlands. We investigated the involvement of children as co-researchers in identifying and analysing themes from interview data presented on video. We conducted the investigation in two phases: Phase 1 consisted of five individual meetings, and Phase 2 of two group meetings. In addition, we reflected on the children's involvement in the analysis process.

Recruitment and sampling

Phase 1

We approached potential co-researchers through national patient support organizations, primary schools, hospitals, social media, and by word-of-mouth. No research experience was required of the participants. Sampling was based on age (9-18 years) and participants were required to be fluent in Dutch because the meetings were held in that language. Initially, seven children volunteered. They each received an information leaflet. Even though recruitment was challenging we managed to achieve our goal of five participants in Phase 1.

Phase 2

We recruited the participants for Phase 2 in collaboration with a specific primary school in Groningen, the Netherlands, where teachers and learners are expected to display an academic mind-set aimed at research and analysis.[20] We invited one class of 15 children of the school's oldest leaners to participate. Ten learners volunteered, one of whom was unable to participate because of illness.

Informed consent

Phase 1

We supplied the potential participants with verbal and written information. We asked them to discuss the study with their parents and to reply by post, e-mail or by telephone. Before the session at the child's home started, he or she read the informed consent form and discussed it with the researcher and a parent. One parent was present throughout the session, but was kindly asked to not interfere with the process. All the children, irrespective of age, were asked to sign the informed consent form to acknowledge that we appreciated their contribution equally. In accordance with the Dutch Medical Research Involving Human Subjects Act, parental consent was obtained in addition to the child's consent.[21] Children were also asked to sign a confidentiality agreement regarding any personal information present in the data

For peer review only - http://bmjopen.bmj.com/site/about/guidelines.xhtml

For peer review only

they analysed. At the end of the session we gave the participants a €10 gift voucher as token of our appreciation of their time and a certificate acknowledging their contribution as co-researchers.

Phase 2

Two adult researchers visited the primary school to meet the learners, their teacher and headmaster, and to introduce the research project. The headmaster agreed to allow the children to participate during school hours. The potential participants received an information leaflet similar to the one given to the participants of Phase 1. We asked them to complete the consent form and the confidentiality agreement at home with a parent, and to return the form to their teacher. At the end of the group meetings we gave the participants a certificate. These children were not given a gift voucher because they participated during school hours.

Data characteristics

Data from the original interview study were analysed by our co-researchers. In addition, the adult researchers took notes during the analyses and written feedback was obtained from the participants before, during, and after participating. In Table 1 we provide additional information on the data collected for the original interview study. Out of a total of 23 interviews two were excluded because they were not videotaped. Another five were excluded because participants and parents had not consented to use the video data.

Table 1. Details of data of the original interview study on children's experiences in medical research

**Study characteristics**	
**Aim**	To explore children's experiences in medical research to obtain recommendations from their perspectives on how to improve children's involvement in research.
**Setting and research team**	Single-centre study conducted by a team of researchers at University Medical Center Groningen, the Netherlands. The research team consisted of an ethicist (EM), paediatrician (EV), and MD/PhD student (ML). All members were trained researchers and/or had previous experience in conducting qualitative research.
**Recruitment and sampling**	Recruitment through health providers from several hospitals, national patient support groups, social media, and by word-of-mouth. Purposive maximum variation sample: children, patients as well as healthy volunteers between 9 and 18 years old who were invited to participate in different types of medical research in the Netherlands and who either took part or declined to take part. The participants had no prior relationships with the members of the research team.
**Informed consent**	Informed consent given by one parent and the child or, in accordance to Dutch law, from 16 years and older by the child only.

For peer review only - http://bmjopen.bmj.com/site/about/guidelines.xhtml

**Data collection**	Twenty three semi-structured, in-depth interviews, lasting between 30 and 100 minutes, with children about their experiences in taking part in medical research, including recommendations for improvement of children's involvement in informed consent procedures and the research itself. A topic guide was developed based on a previous study in the United Kingdom. Interviews performed by ML took place at children's homes and were recorded on audio or video, transcribed verbatim, and returned to the participants. No comments from participants were received. Data collection continued until we reached data saturation of main themes.
**Ethical approval**	The conclusion of the Medical Ethics Review Board of the University Medical Center Groningen was that this study, no. M16.192386, 10 May 2016 fell beyond the scope of the Dutch Medical Research Involving Human Subjects Act .

The co-researchers received a brief interactive introduction to paediatric research and to the original interview study. They were asked to identify the main topics in video interviews and to summarise them on mind maps *(A type of diagram with lines and circles for organising information so that it is easier to use or remember*).[22, p1] To prevent the children from becoming 'little adult researchers' we did not train them extensively. Extensive training would also have been more time consuming for both the children and the researchers.

In Phase 1 the five co-researchers collaborated with the coordinating researcher (PPIA1 – PPI adult researcher 1) in a one-on-one session to identify the main themes in five different interviews. Together they watched a video of between 25 to 45 minutes of another young person and discussed the emerging themes. This took place at the participants' home and lasted between two and three and a half hours. During Phase 1 the adult researcher spent a total of eleven hours travelling to and from co-researchers homes.

During the two group meetings in Phase 2, we explored in detail two themes that had been identified during Phase 1. For this purpose we compiled two five-minute videos from fragments of several interviews from the dataset of the original study. The group meetings took place at a local primary school and lasted approximately two and a half hours. Travelling time for adult researchers was two hours, including the introductory meeting when we handed out the information sheets. In addition to travelling time, the time investment of adult researchers was approximately five hours. This included the time spent preparing the instructions and making the compilation videos. The material costs of the project were low and the travelling costs minimal.

For peer review only - http://bmjopen.bmj.com/site/about/guidelines.xhtml

The aim of the analysis process in both phases was to identify the main themes in the video through open, unstructured discussions with the child co-researchers. To facilitate interaction and discussion the co-researchers and the adult researchers could pause the video at any time. They took notes of what they thought the interviewee found important. PPIA1 and PPIA2 allowed moments of personal reflection, but were on guard for potential intertwining of co-researchers' personal experiences with interviewees' experiences. After watching the video the co-researchers drew a mind map depicting their interpretation of the connection between different themes.[22] The mind maps were drawn on A3-sized sheets of paper, using different sizes of sticky notes, and coloured pens. Besides, the participants were free to use materials of their own choice. The researcher asked the participants questions about the importance of certain themes, the identification of overlapping themes, and the reason why they had chosen a certain theme. The participants led the discussion and made the final decision in case of a disagreement about a theme.

Data collection and analysis

Before the study actually commenced, we asked the child co-researchers why they would like to take part. The adult researchers took notes of how participants fulfilled their role as co-researcher, and how the participants reflected on this process. Child co-researchers completed a feedback form after the analysis (Table 2). In addition, we briefly evaluated the process orally. All meetings were recorded on audio tape.

We performed a thematic analysis using a framework approach.[23] Familiarisation and initial theme identification was done by PPIA1 (ML) and discussed with EM (individual meetings) and PPIA2 (group meetings) based on the audio tape, notes, and the participants' written feedback. Some themes, such as time investment, were identified in advance from the literature, others were derived from the data. Themes were refined and conceptualised during regular meetings with the research team and any disagreements were discussed and a final decision reached by consensus. Because the sample for this exploratory study was relatively small, we did not aim for data saturation.

Table 2. Feedback form

1. Did you understand beforehand what your role was in the project? (No / a little / yes)<br>2. How could we improve the information about working as a co-researcher?	6. Would you recommend other children to become co-researchers? Why?<br>7. How would you rate your time investment? (Too long, adequate, too short)

For peer review only - http://bmjopen.bmj.com/site/about/guidelines.xhtml

3. What was it like for you to work as a co-researcher?<br>a. positive aspects<br>b. points of improvement<br>4. Did you learn anything from being a co-researcher? If so, what did you learn?<br>5. Would you like to be a co-researcher more often?	8. a. What did you think of the EUR 10 gift voucher? (Phase 1)<br>b. What did you think about participating in this project during school hours? Why? (Phase 2)<br>9. Do you have suggestions for improving this evaluation form?

Patient and public involvement

We explored the feasibility of an approach whereby children were involved in qualitative data analysis, both in helping adult researchers to identify themes from original data as in exploring the themes in more detail.

RESULTS

Participant characteristics

Fourteen children, eight girls and six boys, participated as co-researchers in this study. Two participants had experience because they had been chronic patients themselves. None of the children had been a co-researcher before. Tables 3 and 4 show the characteristics of the child and adult participants.

Table 3. Child participant characteristics

Characteristics of child co-researchers	N* (%)**
Sex	
Girl	8 (57)
Boy	6 (43)
Age	
10 years	1 (7)
11 years	10 (71)
12 years	2 (14)
13 years	0 (0)
14 years	1 (7)
School attended	
Primary School	13 (93)
Secondary School	1 (7)
Hospital/Disease experience (lived experience)	
Currently suffering from a disease	2 (14)
Hospitalization or minor surgery in the past	6 (43)
Family member(s) who suffer from a disease	2 (14)
None	4 (29)
Research experience as participant	
Yes	2 (14)
No	12 (86)
Experience as co-researcher	
Yes	0 (0)
No	14 (100)

For peer review only

* Number of child participants; N = 14
** Rounded to nearest whole number

Table 4. Adult participants characteristics

Participant	Sex	Experience illness /hospital	Research experience (participating in research)	Research experience (performing research)
PPIA1	Woman	As a medical student. Graduated as medical doctor in August 2019.	Yes. Participant in two large cohort studies for several years.	Training and experience in qualitative research for PhD.
PPIA2	Woman	As a medical student. Started internships in September 2019.	No previous experience in medical research participation.	Trained in qualitative research as a former psychology student.

Reflection and evaluation of the involvement process

The results can be divided into five main themes: (1) understanding the study procedures, (2) empowerment, (3) reflection on health and illness, (4) interest in the bigger picture, and (5) reflection on time investment. The results of the feedback form are summarised in Table 5.

Table 5. Summary of written feedback

Theme (question from Table 2)	Summary of written feedback	N* (%)**
**Understanding the study procedures** (1,2,9)	*Understanding their role as co-researcher before start****	
	No	0 (0)
	A little	12 (86)
	Yes	2 (14)
	Suggestions for improving the information about working as co-researcher	
	Don't know	
	Everything was clear	4 (29)
	Use fewer difficult words	5 (36)
	Explain that we had to take notes and create a mind map	4 (29)
		1 (7)
	Suggestions for improving the feedback form of the co-researcher project	
	Adding a question about the overall experience	1 (7)
	No recommendations	13 (93)
**Empowerment** (3-6)	*Positive experience as co-researcher*	
	Fun	14 (100)
	Interesting	4 (29)
	Helping other children	1 (7)
	Learning something new	1 (7)
	Time investment was okay	2 (14)
	Receiving a certificate	1 (7)
	Points of improvement for co-researcher project	
	No points of improvement	12 (86)
	Shorter interviews	1 (7)
	The project should take the whole school day (instead of a half one)	1 (7)
	Lessons learnt from being a co-researcher	
	Taking notes	2 (14)
	Critical thinking and listening	4 (29)
	About a medical condition	2 (14)
	About doing research	2 (14)

For peer review only

	About how children think and feel about research<br>That children think differently from adults<br>That it is fun and that you learn a lot<br>Not really	2 (14)<br>1 (7)<br>1 (7)<br>1 (7)
	Would like to be co-researcher more often including reason<br>Yes, because it's fun<br>Yes, because it's interesting<br>Yes, because I like to help people<br>Yes, I know what to expect now<br>Yes, if it doesn't hurt<br>It is fun, but depends on how much time I have	 12 (86)<br>5 (36)<br>1 (7)<br>1 (7)<br>1 (7)<br>1 (7)
	Would you recommend others to become co-researcher?<br>Yes, because it's (super) fun<br>Yes, because it's interesting/you learn something from it<br>Yes, because you receive a gift voucher<br>Yes, because you can help other people<br>Yes, because you get sweets<br>Yes, but it depends on whether it suits them	 9 (64)<br>7 (50)<br>1 (7)<br>2 (14)<br>1 (7)<br>1 (7)
**Time investment** (7,8b)	*Rating of time investment****<br>Too long<br>Adequate<br>Too short	 1 (7)<br>12 (86)<br>1 (7)
	*Thoughts on having this project during school time (Phase 2, n=9)*****<br>Fun/good, because you didn't have to do schoolwork<br>Fun/good, because you don't miss free time after school<br>Don't mind	 6 (67)<br>4 (44)<br>1 (11)
**Compensation** (8b)	*Thoughts on receiving a gift voucher (Phase 1, n=5)*****<br>Fun/good<br>Not necessary<br>Creative	 5 (100)<br>2 (40)<br>1 (20)

* Number of child participants; N = 14. Some participants provided more than one answer
** Rounded to nearest whole number
*** The feedback provided is based on a multiple choice question
**** Calculation based on a sub-selection of total participants, because children took part in a different phase

1. Understanding of study procedures

When we asked the participants in the group meetings what they expected of the session, they remembered that the main idea was '*doing research about research.*' Nonetheless, not everyone remembered the details such as whether photographs would be taken. Even though the information was especially written with children in mind, the participants recalled that it was mostly read and signed by their parents, and had not always been discussed with them. In the case of the co-researchers in the individual meetings this was different. Here the researcher and the parents were present and encouraged the children to complete the form themselves. Parents were available in the background in case their help was needed.
Most children reported that they had a general idea about what their role was in the project. We explained their role to them in detail during the actual project. One of them explained:

For peer review only - http://bmjopen.bmj.com/site/about/guidelines.xhtml

'I already understood it but once you are doing it you understand it [better].' (Girl, individual meeting)

2. Empowerment

By involving children as co-researchers they gained knowledge, they learnt new skills, and they became more confident in fulfilling their role during the analysis process. This was mostly because they enjoyed the new experience of contributing to research or helping others. Children felt empowered by working as a co-researcher. One of them explained it as:

'It actually felt a bit like I was a researcher myself.' (Girl, group meeting)

They reported gaining new knowledge about certain health matters and they realised what it is like to be ill. They learnt how to do research, how to think critically, and how to take notes. Here are some representative answers given:

'You have to think carefully before you draw conclusions.' (Boy, group meeting)

'A bit about how ill children felt afterwards [after participating in research].' (Girl, group meeting)

All the participants were positive about the idea of being a co-researcher more often, mostly for similar reasons as for wanting to take part in the first place:

'Yes, it was fun, relaxed, and instructive.' (Girl, group meeting)

One participant, the only one attending secondary school, mentioned it would depend on whether he had the time to take part because of homework and sports activities in his free time. All participants reported that they would recommend others to become co-researchers, though one participant acknowledged that it might not suit everyone. She mentioned that some children might not enjoy it or might not have the skills to do such work.

During the analysis process in Phase 1, the co-researchers grew noticeably more confident as time progressed. The adult researcher retreated to the background and stimulated the co-researchers to take the lead, which most of them did eventually. One participants actually

For peer review only - http://bmjopen.bmj.com/site/about/guidelines.xhtml

For peer review only

asked the researcher questions about her observations, instead of the other way around. Most participants, however, needed some form of structuring support from the adult researcher throughout.

During the group meetings the support needed from the adult researcher was different. The co-researchers needed more structuring because of group dynamics. Confusion due to competing voices ensued, and shyer children tended to not be heard. Interestingly, the two groups chose different ways of translating their notes to the mind map. In one group the adult researcher noticed a clear distinction in participants' role preferences, and the co-researchers divided the roles between themselves. Some preferred an executive role, such as writing down themes on the 'sticky notes', while others preferred to simply express their ideas and to play a more coordinating role. Some found it difficult to summarise their notes and suggested first underlining important notes:

> *'We could also just first underline what we think is important.'* (Girl, group meeting)

The co-researchers in the other group together decided that they all wanted to write down their own notes on 'sticky notes' and to put them all on the mind map. The outcome was a mind map that displayed different topics as well as providing insight into how important the individuals thought a certain topic was by the number of 'sticky notes' on the same topic. Others only needed a bit more time and space to find their own role.

It was challenging for the adult researchers to not provide answers themselves when the participants indicated that they did not know how to proceed with the analysis. By repeating or rephrasing their question and by acknowledging that they were doing the right thing, the adult researcher could reinforce the children. Both adult researchers were surprised by the co-researcher's achievements. Throughout the project the co-researchers displayed the ability to identify themes and to visualise them in mind maps, underlining the feasibility of this approach and its value for interpreting data.

The researchers learnt a great deal from involving children in the analyses because the participants were very open and direct in their feedback. If they did not understand something, for example, if a question was not clearly formulated, or if they did not understand medical jargon such as 'treatment protocol', they said so immediately.

For peer review only - http://bmjopen.bmj.com/site/about/guidelines.xhtml

Whereas the adult researchers tended to generalise findings, the co-researchers stuck more to the original data. One topic, for example, dealt with recommendations to researchers for improving young people's experiences when participation in medical research. The interviewees mentioned issues like making hospital visits more enjoyable and gave concrete suggestions. All the researchers, adults and children alike, started from the original data, but there appeared to be a difference in analysis. The concrete suggestions in the children's analyses remained and their recommendations were therefore emphasised, while the adults generalised them into 'ways to brighten up the visits'. The co-researchers brought the adults back to the basics that were important to children.

3. Reflection on health and illness

The participants empathised with the young people who shared their experiences in the video. They wondered whether they were still ill or asked if they still lived and hoped they were all right. The following is a representative example:

> *'None of these children [in the video] is deadly ill, right?'* (Girl, group meeting)

The adult researcher explained the different illnesses the interviewees had, and mentioned that some of them had been critically ill, for example, with leukaemia, but that they were stable at the time they were interviewed.

The co-researchers asked questions about the illnesses and what the consequences might be for the lives of the children such as two siblings with a hereditary condition. They also shared their own experiences, for example, about relatives who had cancer.

4. Interest in the bigger picture

Even though we only involved the participants in the analysis stage of the main interview study, they were well aware that this was part of a bigger study. They asked about the other participants and previous experiences with involving children in research analysis, and wondered whether we would show the mind maps they had made to the interviewees. Some of the questions they asked were:

For peer review only - http://bmjopen.bmj.com/site/about/guidelines.xhtml

For peer review only

'Have you been to children's homes?' 'Are you also doing this [project] at other schools?' 'How many times have you done this?' (Several co-researchers, boys and girls, group meetings, not clearly identifiable from the audio)

Hearing that they were the first group of children that participated as co-researchers in this project made them feel special. They expressed the wish to receive the final results and hoped we would do this project again. The co-researchers had a broader interest than just fulfilling their role as co-researchers. They also asked personal questions, such as why the adult researchers did this research project and whether it was part of their university training. They also acknowledged and enjoyed helping the adult researchers with their research:

'It is, of course, good for you [adult researchers] that we participate so that you can continue doing research about research of the research.' (Girl, group meeting)

5. Reflection on time investment

Most children said the time investment was appropriate. One child in the group meetings reported that a shorter meeting would have been better because some children became distracted:

'*Because at the end we were chatting a bit, [we got] distracted.'* (Boy, group meeting)

This was also observed by the adult researchers. Another participant, however, reported that he would have like the session to last longer because he really liked it and did not want to return to his normal schoolwork. The Phase 2 participants all reported that it was an interesting and fun alternative to normal school tasks. They thought it was good to do the project during school time because this way they would not miss out on any free time. One of the participants explained this:

'It was fun because you didn't have to work, and if it hadn't been during school it was inconvenient.' (Girl, group meeting)

From the adult perspective, time was invested in recruitment, developing material to introduce and explain the procedure, and thinking about how to best involve children. The actual analysis with co-researchers was time-intensive and lasted longer than we had expected.

For peer review only - http://bmjopen.bmj.com/site/about/guidelines.xhtml

Nevertheless, the time invested was considered reasonable given the empowerment of children, their learning new skills, and their views on our data provided additional insight, which is promising for the interpretation of our interviews.

DISCUSSION

Little evidence is available on how to involve children in research.[11] In this paper we describe how we involved relatively young children in the analysis of medical research interviews analysis using a two-phase approach. We deployed various strategies to avoid a tokenistic approach, to address challenges regarding time management, and to empower children during the process.

Two-phase approach

Involving relatively young children, aged 10 to14, in our interview analyses was a challenging process. Our aim was to limit preselection of data by adults by introducing one-on-one meetings during which research interviews were analysed by young co-researchers. This approach could be considered an extension of Best's 'participatory theme elicitation' method.[19] Even though the sessions lasted longer than originally intended, the fact that we could focus on one individual worked to our advantage. In our opinion the project benefitted from the fact that the individual meetings were held at the children's homes, which constituted a safe and familiar environment. This confirms findings from Dovey-Pearce and colleagues who highlighted the importance of face-to-face meetings to establish relationships.[18] The themes identified during Phase 1 were explored in detail during group meetings in Phase 2. Working with a number of co-researchers in this phase improved the rigor of the qualitative analysis. In addition, there was an unexpected positive result for the co-researchers who were classmates. Their bond was strengthened by their shared research experience and by reflecting on health and illness together. The two-phase approach enabled us to achieve our research goals and to empower our co-researchers, while keeping within reasonable the time limits. This applied to adult and child researchers alike.

Use of videos in analysis

Our aim was to involve child co-researchers in interview analyses in an effective and efficient way. Data analysis in qualitative research is often lengthy process involving large quantities of text. Locock and colleagues reported that young people reading through the transcripts was tedious and inefficient. They concluded that it was more effective to discuss the data rather than digging into detailed transcripts.[9] We decided to explore other ways of involving

For peer review only - http://bmjopen.bmj.com/site/about/guidelines.xhtml

young co-researchers in interview analyses. Visuals such as photographs, drawings, or mapping methods are often used in collaborations with young children to collect data about children's views on, for example, what they value in their lives.[24] Darbyshire and colleagues reported that using a variety of qualitative visual techniques is helpful for engaging children in research. It also provides a good way for children to express their views. There is, however, a problem with using visuals as a participatory method rather than in analysis, as Darbyshire and colleagues pointed out: *'...having children take photographs and then having only adults "interpret" (or possibly misinterpret) them is potentially an adultist approach to research on children that we sought to avoid.'*[25] For this reason we used videos in the analysis stage to visualise the interview data to be analysed. Our study confirmed the benefits expressed by Darbyshire and colleagues. The co-researchers enjoyed the creative process of developing the mind map and the videos helped them understand and empathise with the interviewees. Using videos rather than transcripts made the process more time-efficient, while preserving the effectiveness of a thematic analysis. Another benefit of videos over transcripts concerned the rigor of the qualitative data analyses. The analysis of interview data is often assumed to start as soon as the interview has been fully transcribed but, even in case of a verbatim transcript including descriptions of vocal emotions such as laughter, the loss of key elements, such as volume of voices and facial expressions, remains. This could present interviewees' experiences in a more abstract way than the original data show.[26, 27] Put differently, by using videos we possibly started the analysis with a more authentic representation of the data.

Additional considerations and further research

Time investment is an important consideration when developing ways of involving children in research analysis. In the method developed by Best and colleagues the analysis is limited to two hours. An additional time investment of four times 90 to 120 minutes is asked of participants for 'capacity building.' In these sessions, young people learn how to design and conduct a study, how to perform qualitative data analyses, and they receive an introduction into the subject matter of their data.[19] We purposely did not train our co-researchers to avoid shaping them to comply with our idea of a qualitative researcher, and to limit time investment. Though we cannot make a comparison, the little training we gave our co-researchers did not seem to have had a negative impact on the result.

For peer review only - http://bmjopen.bmj.com/site/about/guidelines.xhtml

In addition to time investment, timing of research meetings should be considered. Many Young People's Advisory Groups generally plan their meetings during school holidays or weekends.[28] The INVOLVE Advisory Group of the United Kingdom's National Institute for Health Research, which supports active public involvement in the National Health Service, public health, and social care research,[29] identified parents and schools as a significant barrier to public involvement during school hours: *…lack of schools' recognition of the value of their work sometimes acts as a barrier to them attending events which involve travel in school hours.*[13, p12] Nevertheless, thanks to a cooperative headmaster, we managed to set up a collaboration with a primary school for Phase 2 of our study, and planned the group meetings during school hours. For our co-researchers in the group meetings, participating during school time was preferable to after school, because they reported that they had busy schedules or had to do homework during their free time.

Our results showed that children tend to include more concrete topics in their analyses, whereas adults analyse data in a more abstract way. This is in line with the cognitive development of children, who transform from concrete to abstract conceptualisation later in adolescence.[30, 31] Consequently, we expect that an evaluation of the data analysis process performed by children, young people, and adults will provide additional interesting insights. Recently, we started testing our two-phase approach with young people aged 16 to 18. They will use this project for a school assignment, thereby creating a situation that is mutually beneficial. If this proves successful, we consider setting up a long-term collaboration with primary schools and secondary schools to optimise collaboration between researchers and children to help decrease the knowledge gap between academia and society.

Study limitations

To test this new approach we started with a small group of young co-researchers. As a consequence, we had to select of interviews from a larger dataset to analyse in Phase 1. We aimed for as much variation as possible within this selection, but we also needed to be pragmatic, regarding the length of the interviews for instance. In addition, in our method children were not involved in making choices about specific quotes used in the results sections. As recruiting co-researchers was challenging, sampling was limited to age and to fluency in the Dutch language. Maximum variation sampling would, however, be preferable to improve reflexivity.

For peer review only - http://bmjopen.bmj.com/site/about/guidelines.xhtml

CONCLUSION

We suggest that the two-phase approach to involving young children in analysing qualitative data is feasible. Its novelty lies in recruiting children to help identify themes from original data before the themes are explored in detail. Thus preselection of data by adults is limited. By combining one-on-one meetings and group meetings the two-phase approach is an effective and efficient way of involving relatively young children in analysing qualitative data. Additional benefits are that children reflect on health and illness in their own lives, they are empowered, and engaged in medical research. We recommend presenting the interview data on videos rather than through transcripts. Videos make it easier for children to understand the data, to empathise with the interviewees, and it limits time investment.

ETHICS APPROVAL

The Medical Ethics Review Board of the University Medical Center Groningen concluded that neither this study (M18.2334032, 24 July 2018) nor our larger study (M16.192386, 10 May 2016) fall within the scope of the Dutch Medical Research Involving Human Subjects Act.

ACKNOWLEDGEMENTS

We thank all the participants in the original interview study and our co-researcher participants in this project. Because this is a meta-analysis of the PPI project, we decided not to invite the co-researchers as co-author of this article. We thank Laura Postma for helping to facilitate the group meetings.

FUNDING STATEMENT

The PPI study itself was a low-cost project. Data collection for the main interview study was funded by the University of Groningen as part of the MD/PhD program of Malou Luchtenberg. This funding body has no role in the design of the study and collection, analysis, or interpretation of data, or in writing the manuscript.

COMPETING INTERESTS STATEMENT

None declared.

DATA SHARING STATEMENT

The original dataset with audiotaped and videotaped interviews is not available for the public on account of privacy concerns.

AUTHOR'S CONTRIBUTION

ML, EM and EV designed the original study. ML was responsible for the data collection in Phase 1 and the group meetings were led by ML (PPIA1). Analysis was performed by ML, checked by EM, and discussed with EV. ML made the first draft of the article, which was subsequently discussed within the team and reviewed by EM and EV. All authors read and approved the final manuscript.

REFERENCES

For peer review only - http://bmjopen.bmj.com/site/about/guidelines.xhtml

For peer review only

1. Hennink M, Hutter I, Bailey A. The nature of qualitative research. Subjectivity and the need for reflexivity. In: Qualitative Research Methods. London: Sage 2011:19-23.
2. Taylor RM, Whelan JS, Gibson F, et al. Involving young people in BRIGHTLIGHT from study inception to secondary data analysis: insights from 10 years of user involvement. *Res Involv Engagem* 2018;4(1):1–14. https://doi.org/10.1186/s40900-018-0135-x
3. Jamal F, Langford R, Daniels P, et al. Consulting with young people to inform systematic reviews: an example from a review on the effects of schools on health. *Heal Expect* 2015;18(6):3225–35. https://doi.org/10.1111/hex.12312
4. Coad J, Evans R. Reflections on practical approaches to involving children and young people in the data analysis process. *Child Soc* 2008 Jan;22(1):41–52. https://doi.org/10.1111/j.1099-0860.2006.00062.x
5. Clavering EK, McLaughlin J. Children's participation in health research: from objects to agents? *Child Care Health Dev* 2010;36(5):603–11. https://doi.org/10.1111/j.1365-2214.2010.01094.x
6. Brady L-M, Preston J. Evaluating the extent and impact of young people's involvement in National Institute for Health Research (NIHR) studies: an assessment of feasibility. 2017 April. http://www.invo.org.uk/wp-content/uploads/2017/06/Impact-of-CYPs-involvement_JLI__GenR.pdf (accessed 01-07-2019)
7. Lundy L, McEvoy L, Byrne B. Working with young children as co-researchers: an approach informed by the United Nations Convention on the Rights of the Child. *Early Educ Dev* 2011;22(5):714–36. https://doi.org/10.1080/10409289.2011.596463
8. Holland S, Renold E, Ross NJ, et al. Power, agency and participatory agendas: a critical exploration of young people's engagement in participative qualitative research. *Childhood* 2010;17(3):360–75. https://doi.org/10.1177/0907568210369310
9. Locock L, Kirkpatrick S, Brading L, et al. Involving service users in the qualitative analysis of patient narratives to support healthcare quality improvement. *Res Involv Engagem* 2019;5(1):1–11. https://doi.org/10.1186/s40900-018-0133-z
10. INVOLVE. What is public involvement in research? *NHS Natl Inst Heal Res* 2015. http://www.invo.org.uk/find-out-more/what-is-public-involvement-in-research-2/ (accessed 10-07-2019)
11. Bird D, Culley L, Lakhanpaul M. Why collaborate with children in health research: an analysis of the risks and benefits of collaboration with children. *Arch Dis Child - Educ Pract Ed* 2013;98(2):42–8. https://doi.org/10.1136/archdischild-2012-303470
12. Preston J, Stones SR, Davies H, et al. How to involve children and young people in what is, after all, their research. *Arch Dis Child* 2019;104(5):494–500. https://doi.org/10.1136/archdischild-2018-315118
13. Sonpal K, Walker E, Swallow V, et al. Report on involving children and young people in research. Southampton: INVOLVE. 2019. https://www.invo.org.uk/wp-content/uploads/2019/06/77620-YPv3-Booklet-WEB.pdf (accessed 01-07-2019)
14. Blackburn S, McLachlan S, Jowett S, et al. The extent, quality and impact of patient and public involvement in primary care research: a mixed methods study. *Res Involv Engagem* 2018;4(1):1–18. https://doi.org/10.1186/s40900-018-0100-8
15. Domecq JP, Prutsky G, Elraiyah T, et al. Patient engagement in research: a systematic review. *BMC Heal Serv Res* 2014;14(89):1–9. https://doi.org/10.1186/1472-6963-14-89
16. Kellett M, Forrest R, Dent N, et al. "Just teach us the skills please, we'll do the rest": Empowering ten-year-olds as active researchers. *Child Soc* 2004;18(5):329–43. https://doi.org/10.1002/chi.807

For peer review only - http://bmjopen.bmj.com/site/about/guidelines.xhtml

17. Chappell P, Rule P, Dlamini M, et al. Troubling power dynamics: youth with disabilities as co-researchers in sexuality research in South Africa. *Childhood* 2014;21(3):385–99. https://doi.org/10.1177/0907568214525427
18. Dovey-Pearse G, Walker S, Fairgrieve S, et al. The burden of proof: The process of involving young people in research. *Helath Expectations* 2019;22:465-474. https://doi.org/10.1111/hex.12870
19. Best P, Badham J, Corepal R, et al. Network methods to support user involvement in qualitative data analyses: an introduction to Participatory Theme Elicitation. *Trials* 2017;18(1):1–14. https://doi.org/10.1186/s13063-017-2289-5
20. Brederoschool. Academische Basisschool 2019. Groningen, the Netherlands. https://brederoschool.openbaaronderwijsgroningen.nl/academischebasisschool/index.html (accessed 09-11-2018)
21. Dutch Medical Research Involving Human Subjects Act (WMO), 26 February 1998. https://wetten.overheid.nl/BWBR0009408/2019-04-02 (accessed 09-04-2019)
22. Cambridge University Press. Meaning of mind map in English. Cambridge Dictionary. 2019. https://dictionary.cambridge.org/dictionary/english/mind-map (accessed 19-09-2019)
23. Smith J, Firth J. Qualitative data analysis: the framework approach. Nurse Researcher 2011;18(2):52–62. https://doi.org/10.7748/nr2011.01.18.2.52.c8284
24. Coad J. Using art-based techniques in engaging children and young people in health care consultations and/or research. *J Res Nurs* 2007;12(5):487–97. https://doi.org/10.1177/1744987107081250
25. Darbyshire P, Macdougall C, Schiller W. Multiple methods in qualitative research with children: more insight or just more? *Qual Res* 2005;5(4):417–436. https://doi.org/10.1177/1468794105056921
26. Hewitt J. Ethical components of researcher-researched relationships in qualitative interviewing. *Qual Health Res* 2007;17(8):1149–59. https://doi.org/10.1177/1049732307308305
27. Alldred P, Gillies V. Eliciting research accounts: re/producing modern subjects. Ethics in Qualitative Research. London: Sage 2002:146–65.
28. Gaillard S, Malik S, Preston J, et al. Involving children and young people in clinical research through the forum of a European Young Persons' Advisory Group: needs and challenges. *Fundam Clin Pharmacol* 2018 Aug;32(4):357–62. https://doi.org/10.1111/fcp.12360
29. National Institute for Health Research. About INVOLVE. https://www.invo.org.uk/about-involve/ (accessed 04-03-2020)
30. Garrison W, Felice ME. Adolescence. In: Carey WB, Crocker AC, Coleman WL, Elias ER, Feldman HM, eds. Developmental-Behavioral Pediatrics (Fourth Edition) 2009:62–73. https://doi.org/https://doi.org/10.1016/B978-1-4160-3370-7.00006-7
31. Christie D, Viner R. Adolescent development. *BMJ (Clinical Research Ed.)* 2005;330:301. https://doi.org/10.1136/bmj.330.7486.301

For peer review only - http://bmjopen.bmj.com/site/about/guidelines.xhtml
